# SARS-CoV-2 impairs the disassembly of stress granules and promotes ALS-associated amyloid aggregation

**DOI:** 10.1007/s13238-022-00905-7

**Published:** 2022-04-06

**Authors:** Yichen Li, Shuaiyao Lu, Jinge Gu, Wencheng Xia, Shengnan Zhang, Shenqing Zhang, Yan Wang, Chong Zhang, Yunpeng Sun, Jian Lei, Cong Liu, Zhaoming Su, Juntao Yang, Xiaozhong Peng, Dan Li

**Affiliations:** 1 Bio-X Institutes, Key Laboratory for the Genetics of Developmental and Neuropsychiatric Disorders, Ministry of Education, Shanghai Jiao Tong University, Shanghai, 200030 China; 2 School of Life Sciences and Biotechnology, Shanghai Jiao Tong University, Shanghai, 200030 China; 3 National Kunming High-level Biosafety Primate Research Center, Institute of Medical Biology, Chinese Academy of Medical Sciences and Peking Union Medical College, Kunming, 650031 China; 4 State Key Laboratory of Medical Molecular Biology, Chinese Academy of Medical Sciences, School of Basic Medicine, Peking Union Medical College, Beijing, 100005 China; 5 State Key Laboratory of Medical Molecular Biology, Department of Biochemistry and Molecular Biology, Institute of Basic Medical Sciences, Chinese Academy of Medical Sciences & Peking Union Medical College, Beijing, 100005 China; 6 Interdisciplinary Research Center on Biology and Chemistry, Shanghai Institute of Organic Chemistry, Chinese Academy of Sciences, Shanghai, 201210 China; 7 University of Chinese Academy of Sciences, Beijing, 100049 China; 8 State Key Laboratory of Biotherapy, Department of Geriatrics and National Clinical Research Center for Geriatrics, West China Hospital, Sichuan University, Chengdu, 610041 China; 9 Bio-X-Renji Hospital Research Center, Renji Hospital, School of Medicine, Shanghai Jiao Tong University, Shanghai, 200240 China; 10 Zhangjiang Institute for Advanced Study, Shanghai Jiao Tong University, Shanghai, 200240 China

**Keywords:** SARS-CoV-2, nucleocapsid protein, stress granule

## Abstract

The nucleocapsid (N) protein of SARS-CoV-2 has been reported to have a high ability of liquid-liquid phase separation, which enables its incorporation into stress granules (SGs) of host cells. However, whether SG invasion by N protein occurs in the scenario of SARS-CoV-2 infection is unknow, neither do we know its consequence. Here, we used SARS-CoV-2 to infect mammalian cells and observed the incorporation of N protein into SGs, which resulted in markedly impaired self-disassembly but stimulated cell cellular clearance of SGs. NMR experiments further showed that N protein binds to the SG-related amyloid proteins via non-specific transient interactions, which not only expedites the phase transition of these proteins to aberrant amyloid aggregation *in vitro*, but also promotes the aggregation of FUS with ALS-associated P525L mutation in cells. In addition, we found that ACE2 is not necessary for the infection of SARS-CoV-2 to mammalian cells. Our work indicates that SARS-CoV-2 infection can impair the disassembly of host SGs and promote the aggregation of SG-related amyloid proteins, which may lead to an increased risk of neurodegeneration.

**Supplementary Information:**

The online version contains supplementary material available at 10.1007/s13238-022-00905-7.

## Introduction

The ongoing pandemic of coronavirus disease 2019 (COVID-19) caused by the severe acute respiratory syndrome coronavirus 2 (SARS-CoV-2) is an international public health emergency. SARS-CoV-2 infection typically causes a contagious respiratory tract illness and occasionally gastrointestinal symptoms (Lee et al., 2020; Mao et al., 2020; Zhou et al., 2020; Zhu et al., 2020). These immediate symptoms would disappear in several weeks as the patients recover from the infection (Soresina et al., 2020; Thevarajan et al., 2020). In the meantime, many evidence have shown that SARS-CoV-2 can also infect human central nervous system (Mao et al., 2020; Zhou et al., 2020; Song et al., 2021) and cause neuroinflammation (Bostanciklioglu, 2020; Gatto and Fernandez Boccazzi, 2020; Hascup and Hascup, 2020; Heneka et al., 2020; Singal et al., 2020), which raise worries about potential long-term effects of COVID-19 especially on the development of neurodegenerative diseases (Gatto and Fernandez Boccazzi, 2020; Heneka et al., 2020; Li et al., 2020; Paniz-Mondolfi et al., 2020; Serrano-Castro et al., 2020). Indeed, virus invasion in neurological system has been linked to the pathogenesis of several neurodegenerative disorders such as Parkinson’s disease (PD), Alzheimer’s disease (AD), and amyotrophic lateral sclerosis (ALS) (Jang et al., 2009; Eimer et al., 2018; Readhead et al., 2018; Bellmann et al., 2019; Marreiros et al., 2020). PD diagnosed after SARS-CoV-2 infection has been also reported (Cohen et al., 2020). However, the relationship between SARS-CoV-2 infection and neurodegeneration requires a lot more evidence to reveal.

SARS-CoV-2 belonging to SARS-related coronaviruses, is an enveloped, positive-sense single-stranded RNA virus with a 30-kb genome (Wu et al., 2020; Zhou et al., 2020; Zhu et al., 2020). Its genomic RNA packages with the nucleocapsid (N) protein to form the so-called nucleocapsid (Lai and Cavanagh, 1997; Saikatendu et al., 2007), which is important for the viral replication and transcription (McBride et al., 2014). The genome of SARS-CoV-2 consists of 14 open reading frames (Orfs) encoding 16 non-structural proteins (Nsp1–16), four structural proteins (spike (S), envelope (E), membrane (M) and nucleocapsid (N)) and nine putative accessory factors (Chan et al., 2020; Wu et al., 2020). A recent mass spectrometry study expressed 26 out of the 29 SARS-CoV-2 proteins in human cells and identified broad interactions between the viral and human proteins involved in biological processes including protein trafficking, translation, transcription and ubiquitination regulation (Gordon et al., 2020). Bioinformatics analysis predicted that the disordered domains of SARS-CoV-2 N protein can engage in π-π intermolecular interactions with host stress granule (SG) proteins, which is crucial for the viral hijacking of host machineries (Moosa and Banerjee, 2020). Recombinant N protein of SARS-CoV-2 exhibits a high ability of liquid-liquid phase separation *in vitro* (Carlson et al., 2020; Chen et al., 2020; Iserman et al., 2020; Savastano et al., 2020; Luo et al., 2021b; Zhao et al., 2021), overexpression of which in human cell lines showed its incorporation into SGs (Savastano et al., 2020; Luo et al., 2021a). These studies indicate the association of N protein with host SGs, while a real scenario of virus infection is lacking. Neither do we know the consequence of the potential invasion of host SGs by SARS-CoV-2.

In this work, we infected mammalian cells with SARS-CoV-2, and observed that N protein but not the other monitored viral proteins incorporated into host SGs. Consequently, the invaded SGs are less dynamic and resistant to disassemble after the removal of stress, but are promoted for clearance upon continuous stress. *In vitro* observations showed cooperative liquid-liquid phase separation (LLPS) of N protein with SG-related amyloid proteins and stimulation of their amyloid aggregation. NMR experiments further characterized the non-specific transient interactions between N protein and SG-related amyloid proteins. The enhancement of amyloid aggregation by SARS-CoV-2 infection was further shown by the exacerbated aggregation of an ALS-associated FUS mutant in cells. In addition, we found that cells without the ACE2 receptor can still be infected by SARS-CoV-2, with a decreased efficiency though. These molecular evidences support that SARS-CoV-2 infection might increase the risk of neurodegenerative diseases.

## Results

### ACE2 is not necessary for SARS-CoV-2 to infect mammalian cells

**Figure 1:**
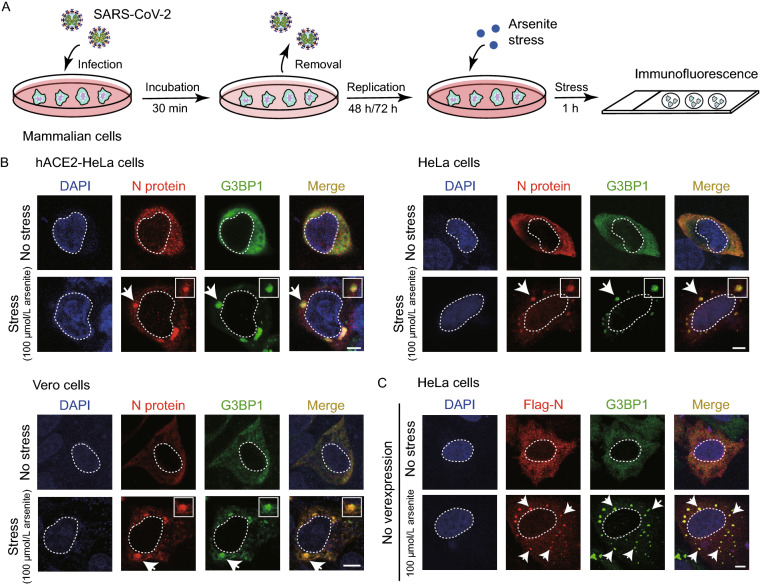
**SARS-CoV-2 infection results in the incorporation of N protein with SGs in mammalian cells**. (A) Schematic workflow of the virus infection and SG induction. (B) Confocal images of mammalian cells infected by SARS-CoV-2. Infected cells were stressed with 100 μmol/L sodium arsenite for 1 h, and stained with DAPI, antibodies for N protein and SG marker proteins G3BP1. Arrows indicate SGs. Scale bar, 5 µm. (C**)** Confocal images of HeLa cells transfected with Flag-tagged N protein (Flag-N). Cells were stressed with 100 μmol/L sodium arsenite for 1 h, and stained with DAPI, anti-flag, and anti-G3BP1. Arrows indicate SGs. Scale bar, 5 µm

To investigate the impact of SARS-CoV-2 infection on host SGs, we first treated mammalian cells including monkey Vero cells and human HeLa cells with SARS-CoV-2 for 30 min (Fig. 1A). Vero cells have the ACE2 receptor (Fig. S1A), which can help the entry of SARS-CoV-2 (Li et al., 2003). After two days for virus replication, expression of viral proteins in Vero cells was detected (Fig. S1A). Although HeLa cells lack ACE2 (Zhou et al., 2020), at a high multiplicity of infection (MOI) of 0.75, SARS-CoV-2 can still infect HeLa cells and the expression of the viral N protein was detected by Western blot (Fig. S1B). However, the expression of Spike was not observed (Fig. S1B), which may reflect poor replication of the virus in HeLa cells. To increase the virus entry efficiency, we also treated HeLa cells overexpressing human ACE2 (ACE2-HeLa cells) with the virus. After 2 days for viral replication, expression of the viral proteins was detected (Fig. S1B). These results indicate that ACE2 is not necessary for SARS-CoV-2 to infect mammalian cells, although it can increase the efficiency.

### N protein enters the host SGs upon SARS-CoV-2 infection

Next, we stressed the infected cells with 100 μmol/L sodium arsenite to induce SGs, and then used immunofluorescence microscopy to monitor the cellular localization of different viral proteins including N protein, S protein, Nsp1, Nsp8 and ORF7α (Fig. 1A). The result showed that N protein but not the others is recruited into the SGs of the mammalian cells (Figs. 1B and S2).

It has been reported that overexpressed N protein of either SARS-CoV-2 or SARS-CoV can invade cellular SGs (Peng et al., 2008; Savastano et al., 2020; Luo et al., 2021a). We confirmed this result by overexpressing recombinant Flag-tagged N protein in HeLa cells, and consistently observed the overexpressed N protein co-localizing with SGs (Fig. 1C). The overexpression results indicate that N protein can incorporate into SGs independent of viral components.

### SARS-CoV-2 infection impairs the self-disassembly of SGs

**Figure 2:**
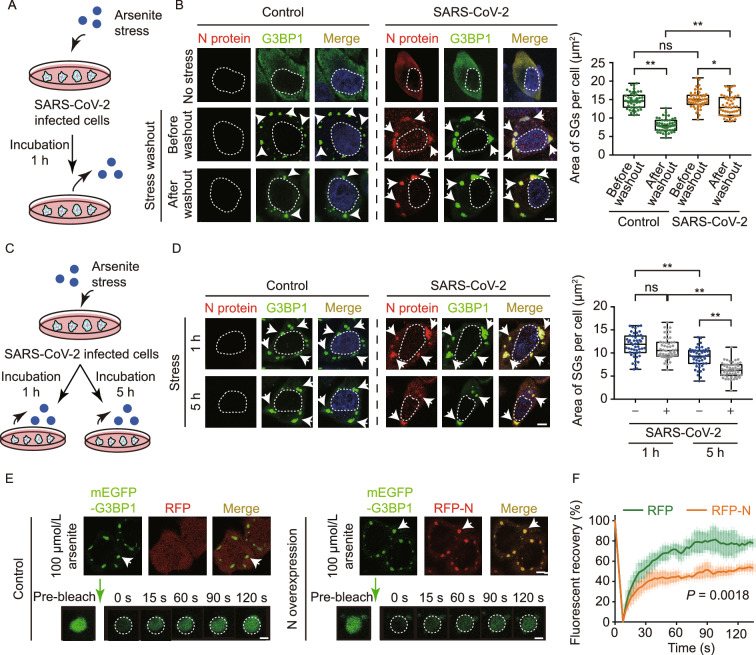
**SARS-CoV-2 infection impairs the disassembly but enhances the cellular clearance of host SGs**. (A) Schematic workflow of the experiment of SG self-disassembly. (B) Confocal images of ACE2-HeLa cells with or without (control) infection of SARS-CoV-2. Cells were stressed with 100 μmol/L sodium arsenite for 1 h, followed by washing out sodium arsenite. Cells were stained with antibodies for viral N protein and SG marker protein G3BP1. Arrows indicate SGs. Scale bar, 5 µm. Quantitative analysis of the images is shown on the right as the area of SGs per cell. Values are means ± SD, *n* > 150 cells from 3 replicates. Student’s *t*-test, ^*^P < 0.05, ^**^P < 0.01; ns, not significant. (C**)** Schematic workflow of the experiment of cellular SG clearance. (D**)** Confocal images of ACE2-HeLa cells with or without (control) SARS-CoV-2 infection. Cells were stressed with 100 μmol/L sodium arsenite for 1 h or 5 h. Cells were stained with antibodies for N and G3BP1 proteins. Arrows indicate SGs. Scale bar, 5 µm. Quantitative analysis of the images is shown on the right as the area of SGs per infected cell. Values are means ± SD, *n* > 150 cells from 3 replicates. Student’s *t*-test, ^*^P < 0.05, ^**^P < 0.01; ns, not significant. (E**)** *In situ* FRAP for SGs in HeLa cells overexpressing RFP-tagged N protein. Cells overexpressing RFP-tag were performed as a control. mEGFP-tagged G3BP1 was co-overexpressed with RFP or RFP-N to fluorescently label SGs. Scale bar, 5 µm. FRAP montages of an SG for each sample are shown. The arrows indicate the action of bleaching. (F**)** Fluorescent recovery curves of the SGs. Data shown are means ± S.D., *n* = 3 individual SGs. Student’s *t*-test

To investigate the influence of SARS-CoV-2 invasion on SG dynamics, we relieved the cellular stress by washing out sodium arsenite in the culture medium (Fig. 2A). Before washout, similar amounts of SGs formed in SARS-CoV-2 infected cells and control cells (not treated with virus) (Fig. 2B), indicating no significant influence of SARS-CoV-2 infection on the assembly of SGs. However, the self-disassembly of SGs was significantly slowed down in the infected cells monitored 60 min post washout (Fig. 2B). Similar phenomena were observed in both HeLa cells and ACE2-overexpressed HeLa cells (Figs. 2B and S3A). We also performed the washout experiment by directly overexpressing N protein in HeLa cells and obtained consistent results with that infected by the virus (Fig. S3B). These results indicate that upon SARS-CoV-2 infection, the invasion of N protein can impair the dynamics, specifically the self-disassembly of SGs.

### SARS-CoV-2 infection promotes cellular clearance of SGs

Previous studies suggest autophagy as a second SG clearance system in addition to self-disassembly (Buchan et al., 2013; Protter and Parker, 2016), and persistent SGs induced by chronic stress are eliminated by autophagy-dependent degradation (Gwon et al., 2021). Indeed, as we stressed the cells for extra h (5 h), we also observed disappearance of SGs (Fig. 2C and 2D), although the involvement of autophagy is not confirmed here. Notably, SARS-CoV-2 infected cells exhibited more severe SG disappearance than the control cells (Fig. 2D). Similar phenomena were observed when overexpressing N protein in HeLa cells (Fig. S4). These results indicate that abnormal SGs due to the incorporation of the viral N protein may be more potent to trigger cellular degradation systems.

### N protein incorporation impairs the liquid-like state of host SGs

To further investigate the influence of N protein incorporation on the liquid-like state of SGs, we performed *in situ* fluorescence recovery after photobleaching (FRAP). We overexpressed G3BP1 with an mEGFP tag as a fluorescent marker of SGs in HeLa cells. FRAP experiment showed that the fluorescent intensity of SGs rapidly recovered about 80% within 2 min after photobleaching reflecting high mobility and liquid-like property of SGs (Fig. 2E). In contrast, as N protein co-overexpressed in the cells, the recovery of fluorescent intensity significantly slowed down to ~50% recovery within 2 min (Fig. 2F). This result indicates that N protein incorporation disrupts the liquid-like state of SGs.

### N protein expedites the maturation of the LLPS of SG-related amyloid proteins

It has been reported that N protein of SARS-CoV-2 has a high ability of LLPS under various conditions (Carlson et al., 2020; Cubuk et al., 2020; Iserman et al., 2020; Luo et al., 2020; Perdikari et al., 2020; Savastano et al., 2020). The LLPS property of N protein has been suggested to play an important role in the viral genome packing in other viruses (Guseva et al., 2020; Monette et al., 2020). We also observed that N protein readily underwent LLPS in the presence of synthetic single-stranded RNA (polyU) (Fig. S5).

**Figure 3:**
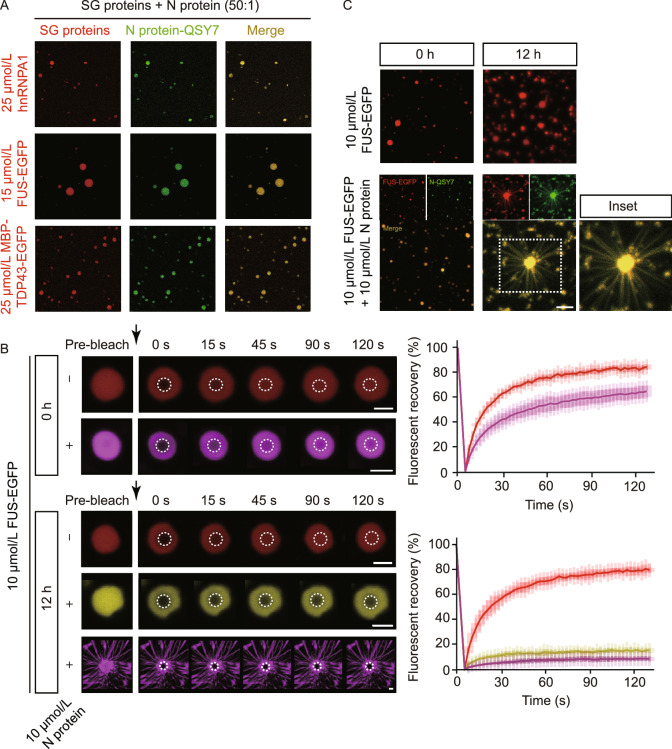
**SARS-CoV-2 N protein co-phase separates with SG proteins and solidifies their liquid-like droplets**. **(A)** Fluorescence images of co-phase separation of N protein with SG proteins hnRNPA1, FUS, and TDP43, respectively. SG protein concentrations and the molar ratio of SG proteins to N protein are indicated. LLPS buffer: 50 mmol/L Tris-HCl, pH 7.5, 100 mmol/L NaCl, 10% PEG 3,350 (no PEG for FUS). EGFP is a fluorescence tag. Alexa-488 and QSY7 are fluorescence dyes. Scale bar, 5 µm. (B**)** FRAP montages of FUS-EGFP droplets (left). The arrows indicate the action of bleaching. Protein concentrations are indicated. Buffer: 50 mmol/L Tris-HCl, pH 7.5, 100 mmol/L NaCl. The droplets are incubated for 0 h and 12 h, respectively. Scale bar, 2 µm. The graphs (right) show the recovery fraction as the function of time. Data shown are means ± SD, *n* = 3. (C**)** Representative images of the morphological changes of FUS-EGFP droplets in the absence or presence of N protein over time. The phase separation condition is the same as (B). A FUS droplet with fibrils growing out is enlarged in the inset. Scale bar, 10 µm

Several RNA-binding proteins of SGs including FUS, hnRNPA1, and TDP43 are meanwhile prone to undergo amyloid aggregation, which is closely associated with neurodegenerative diseases such as ALS and frontotemporal dementia (FTD). These proteins also have a high ability of LLPS (Molliex et al., 2015; Patel et al., 2015). To investigate the influence of SARS-CoV-2 N protein on the phase transition of these SG-related amyloid proteins, we added the viral N protein to the LLPS solution of human FUS, hnRNPA1, and TDP43, respectively. Fluorescent microscopic imaging showed that N protein spontaneously condenses in the droplets formed by the SG proteins (Fig. 3A), which is consistent with a previous report showing that N protein can co-phase separate with FUS, TDP43, and hnRNPA2 (Perdikari et al., 2020).

Moreover, we observed that in the presence of N protein, the fluorescent intensity of EGFP-tagged FUS (FUS-EGFP) recovered markedly slower than that in the absence of N protein (Fig. 3B). After incubation for 12 h, the shape of the droplets was apparently less round than that at 0 h (Fig. 3B). FRAP showed that these droplets nearly lost the mobility and were hardly recovered after bleaching (Fig. 3B). Similarly, N protein also impairs the liquid-like nature of the hnRNPA1 and TDP43 droplets by FRAP experiments (Fig. S6). These data indicate that the involvement of N protein accelerates the liquid-to-solid maturation process of these SG proteins, which is in line with the impaired dynamics of SGs in cells stated above.

### Non-specific transient interactions between N protein and the LC domains of SG-related proteins

**Figure 4:**
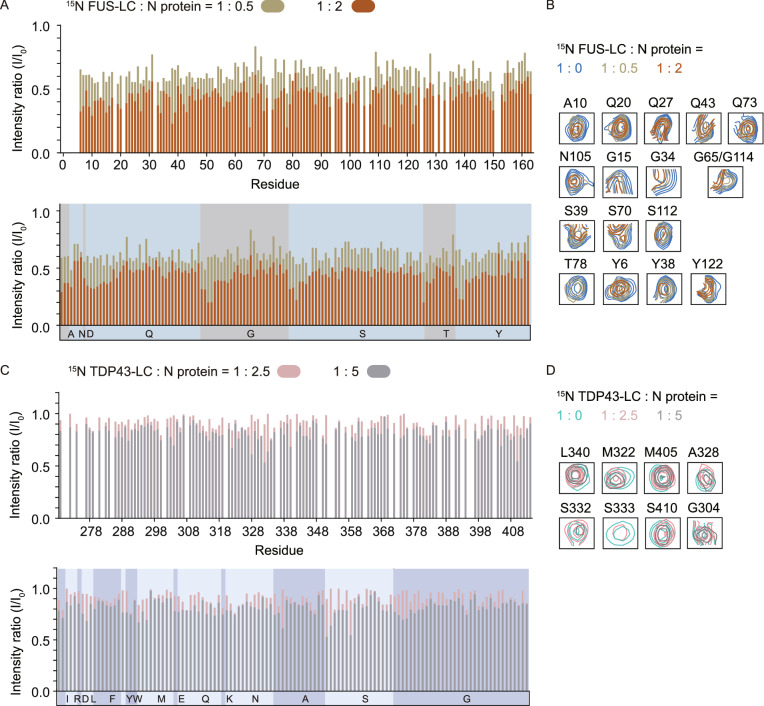
**SARS-CoV-2 N protein non-specifically interacts with LC domains of FUS and TDP-43**. (A) Residue-specific intensity changes of signals in the 2D ^1^H-^15^N HSQC spectra of 25 μmol/L ^15^N-labeled FUS-LC in the presence of N protein at molar ratios (FUS-LC : N) of 1:0.5 (green) and 1:2 (orange). The x axis for the spectra on the top is shown according to the residue numbers; that at the bottom is according to the amino acid composition. Residue signals that dropped over 70% are shown in (B). (C) Residue-specific intensity changes of signals in the 2D ^1^H-^15^N HSQC spectra of 20 μmol/L ^15^N-labeled TDP43-LC in the presence of N protein at molar ratios (TDP43-LC : N) of 1:2.5 (pink) and 1:5 (grey). Residue signals that dropped over 30% are shown in (B)

We next used NMR spectroscopy to study the molecular mechanism underlying the interaction between N protein and the SG proteins. The 2D ^1^H-^15^N HSQC spectra of ^15^N-labeled FUS low complex domain (FUS-LC) and ^15^N-labeled TDP43-LC showed that both proteins adopted an intrinsically disordered conformation with a narrow chemical shift dispersion in the ^1^H dimension (backbone amide resonances within 7.8–8.8 ppm) (Fig. S7), which is consistent with the previous reports (Burke et al., 2015; Conicella et al., 2016; Liu et al., 2020; Gu et al., 2021a). We then used unlabeled N protein to titrate ^15^N-labeled FUS-LC. The HSQC spectra of FUS-LC showed a global decrease of signal intensities in a concentration-dependent manner (Figs. 4A, 4B and S7B), indicating direct interaction between N protein and FUS-LC. However, no specific region or residue type showed significant intensity decrease compared to others, and residues of FUS-LC suffered an overall ~40% intensity decrease at the substoichiometric molar ration of 1:0.5 (FUS-LC:N) (Fig. 4A), implying that the interaction between N protein and FUS-LC is non-specific. Similarly, titration of N protein to TDP43-LC resulted in concentration-dependent attenuations of the signal intensities in the HSQC spectra of TDP43-LC (Figs. 4C, 4D and S7D). The interaction between N protein and TDP43-LC also appears non-specific, since the residues of TDP43-LC generally showed a ~20% intensity decrease at the molar ratio of 1:5 (TDP43-LC:N) (Figs. 4C, 4D and S7D). Unfortunately, we did not get a good NMR spectrum of hnRNPA1-LC, in which only a few broadening crosspeaks could be detected at our conditions. Taken together, these results indicate that N protein may commonly bind the SG proteins via nonspecific weak interactions to the LC domains of the SG proteins.

### N protein stimulates the aggregation of SG-related amyloid proteins

As we incubated N protein with FUS for 12 h, we observed that some LLPS droplets became spiky with fibrils growing out of the droplets (Fig. 3B and 3C). FRAP experiment showed that the fluorescence of these spiky droplets can hardly recover after bleaching, which confirms the solid nature of these droplets (Fig. 3B).

**Figure 5:**
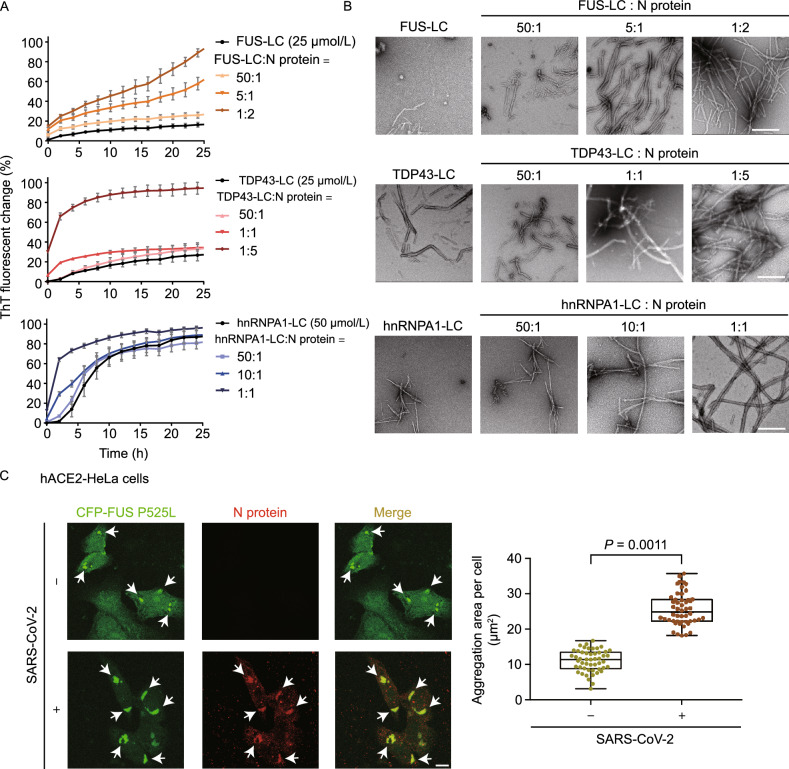
**SARS-CoV-2 N protein promotes the amyloid aggregation of SG proteins**. (A) ThT kinetic assays for amyloid fibril formation of the LC domains of FUS, TDP43, and hnRNPA1. Protein concentrations and molar ratios are indicated. Buffer for FUS-LC and hnRNPA1-LC amyloid formation: 50 mmol/L Tris-HCl, pH 7.5 and 100 mmol/L NaCl. Buffer for TDP43-LC amyloid formation: 50 mmol/L Bis-Tris, pH 6.5 and 100 mmol/L NaCl. Data correspond to mean ± SD, *n* = 3 independent replicates. (B**)** TEM images of samples in (A) at the end time point. Scale bar, 500 nm. (C**)** Confocal images of CFP-FUS P525L aggregation puncta in ACE2-HeLa cells infected by SARS-CoV-2. Cells without treatment of the virus are used as a control. FUS P525L is visualized by CFP fluorescence. N protein is immunostained with anti-N. The arrows indicate FUS P525L aggregates. Scale bar, 5 μm. Quantitative analysis of the aggregation area per cell in the imaging data is shown on the right. Values are means ± SD, *n* > 150 cells from 3 replicates. Student’s *t*-test

To further examine the influence of N protein on the amyloid fibril formation of FUS, hnRNPA1, and TDP43, we performed the ThT fluorescence assay and negative-staining transmission electron microscopy (TEM). Since the LC domains of FUS, hnRNPA1, and TDP43 are the amyloid-forming core sequences of these proteins (Johnson et al., 2009; Kato et al., 2012; Kim et al., 2013), we incubated N protein with the LC domains of these three SG proteins, respectively. The result showed that the presence of N protein markedly enhanced the ThT intensities of the fibril-forming samples, and shortened the lag time of the ThT kinetic curves in a dose-dependent manner (Fig. 5A). TEM imaging confirmed amyloid fibril formation in these samples (Fig. 5B). In addition, we confirmed that N protein under the examined conditions does not form amyloid fibrils (Fig. S8). These results indicate that N protein can generally stimulate the phase transition of the SG-related amyloid proteins into amyloid aggregation.

### SARS-CoV-2 infection promotes amyloid aggregation of ALS-related FUS mutant in cells

We next sought to investigate SARS-CoV-2 infection on protein amyloid aggregation in cells. We used a cell model that overexpresses FUS with P525L mutation, a mutation found in ALS that disrupts the nuclear localization of FUS and results in FUS accumulation in the cytoplasm (Kwiatkowski et al., 2009; Dormann et al., 2010; De Santis et al., 2019). We first transfected ACE2-overexpressed HeLa cells with CFP-fused FUS P525L. Aggregation of FUS P525L in cells can be probed CFP fluorescence and an amyloid dye—pFTAA (Klingstedt et al., 2013; Qamar et al., 2018). Next, we treated the cells with SARS-CoV-2 and observed that the viral N protein colocalizes with FUS P525L aggregates (Fig. 5C). Notably, comparing with the control cells (no virus infection), the aggregation of FUS P525L significantly increased upon virus infection (Fig. 5C). We also observed the same enhancement of FUS P525L aggregation by using SARS-CoV-2 infected HeLa cells (Fig. S9A) and HeLa cells with overexpressed N protein (Fig. S9B). These data strengthen the potential consequence of SARS-CoV-2 infection in stimulating protein amyloid aggregation in the host cells.

## Discussion

**Figure 6:**
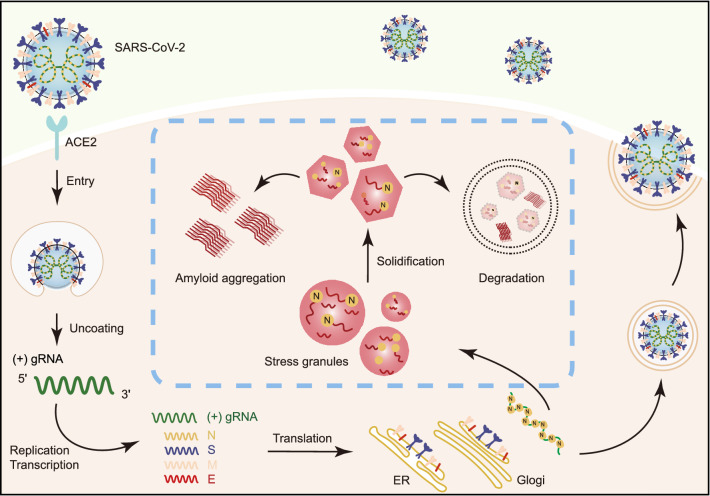
**Schematic diagram for the interplay between SARS-CoV-2 and host SGs**. During SARS-CoV-2 replication in host cells, the viral N protein enters the host SGs and directly interacts with SG-related amyloid-forming proteins (e.g., FUS, hnRNPA1 and TDP43), which stimulates the liquid to solid phase transition (amyloid formation) of these host proteins. Inefficient maintenance of proteostasis may result in accumulation of the pathological amyloid fibrils and development of neurodegeneration

Virus infection has been found to play an important role in the pathogenesis and clinical onset of human neurodegenerative diseases (Jang et al., 2009; Eimer et al., 2018; Readhead et al., 2018; Bellmann et al., 2019; Marreiros et al., 2020). Recent studies have shown that proteins of SARS-CoV-2 accumulate in the brain tissues of both transgenic mice and patients who died from COVID-19 (Matschke et al., 2020; Song et al., 2021). It has also been reported that SARS-CoV-2 can infect and replicate in astrocytes (Crunfli et al., 2021). Clinical correlation of SARS-CoV-2 infection and PD onset has also been reported. These observations raise the concern of neurodegeneration as a long-term consequence of COVID-19 (Bostanciklioglu, 2020; Gatto and Fernandez Boccazzi, 2020; Hascup and Hascup, 2020; Heneka et al., 2020; Singal et al., 2020). The answer for this concern is important for our treatment and policy to COVID-19. Our work demonstrates that as SARS-CoV-2 infects the host cells, it has a strong potential to stimulate the amyloid aggregation of host proteins, which provides molecular evidence for the role of SARS-CoV-2 in triggering neurodegeneration (Fig. 6). During this process, the viral N protein is a major player. N protein can interact with a wide spectrum of SG proteins including FUS, TDP43, hnRNPA1, hnRNPA2, G3BP1, and G3BP2 as reported previously and in this work (Kaur and Lal, 2020; Luo et al., 2020; Moosa and Banerjee, 2020; Perdikari et al., 2020). Our NMR data showed that N protein non-specifically interacts with the LC domains of FUS and TDP43. Given that the SG-related RNA-binding proteins generally contain intrinsically disordered sequences, N protein may interact with other SG proteins via a similar mechanism. Direct interactions with various SG proteins underlie the partition of N protein into SGs; however, whether this process is active or passive is obscure. SG formation is part of the antiviral responses of cells, which can assemble in response to viral infection and function to sequester host and viral mRNAs and proteins (Onomoto et al., 2012; Jain et al., 2016). On the other hand, N protein incorporation may hijack host SGs and alter their attributes. In addition, the viral genomic RNA, that assembles with N protein to form nucleocapsid, may facilitate the SG incorporation of N protein. Indeed, we observed that single-stranded RNA lowered the critical concentration of N protein for LLPS (Fig. S5). Note that in our experiments, we did not observe viral infection-triggered SG formation in either human HeLa cells or monkey Vero cells, which may be caused by reasons such as cell sensitivity, virus infection titer and time.

Many evidences have indicated that disruption of SG dynamics is closely associated with neurodegenerative diseases such as ALS and FTD (Molliex et al., 2015; Duan et al., 2019; Wolozin and Ivanov, 2019; Zhang et al., 2020). Our work demonstrates that SARS-CoV-2 infection can impair the dynamics of SGs and promote amyloid aggregation of SG-related proteins. In addition, our data show that HeLa cells, that same as neurons, lack the ACE2 receptor, can be infected by SARS-CoV-2 (Fig. S1B). Consistently, recent studies have identified several other receptors that may contribute to ACE2-independent cell entry of SARS-CoV-2 (Gao et al., 2020; Amraei et al., 2021; Chen et al., 2021; Gu et al., 2021b). These indicate that neurons are likely to be infected by SARS-CoV-2 as the virus invades the human brain. Our work provides molecular evidence for the increased risk of neurodegeneration after SARS-CoV-2 infection, and suggests paying a special attention to the incidence of neurodegenerative diseases in aged people under the current circumstances of ongoing widespread of SARS-CoV-2.

## Supplementary Information

Below is the link to the electronic supplementary material.

Supplementary file1 (PDF 1645 kb)

## References

[CR1] Amraei R, Yin W, Napoleon MA, Suder EL, Berrigan J, Zhao Q, Olejnik J, Chandler K, Xia C, Feldman J, et al (2021) CD209L/L-SIGN and CD209/DC-SIGN act as receptors for SARS-CoV-2. bioRxiv 10.1021/acscentsci.0c01537 PMC8265543 34341769

[CR2] Bellmann J, Monette A, Tripathy V, Sojka A, Abo-Rady M, Janosh A, Bhatnagar R, Bickle M, Mouland AJ, Sterneckert J (2019). Viral infections exacerbate FUS-ALS phenotypes in iPSC-derived spinal neurons in a virus species-specific manner. Front Cell Neurosci.

[CR3] Bostanciklioglu M (2020) Severe acute respiratory syndrome coronavirus 2 is penetrating to dementia research. Curr Neurovasc Res. 10.2174/1567202617666200522220509 32442082

[CR4] Buchan JR, Kolaitis RM, Taylor JP, Parker R (2013). Eukaryotic stress granules are cleared by autophagy and Cdc48/VCP function. Cell.

[CR5] Burke KA, Janke AM, Rhine CL, Fawzi NL (2015). Residue-by-residue view of in vitro FUS granules that bind the C-terminal domain of RNA polymerase II. Mol Cell.

[CR6] Carlson CR, Asfaha JB, Ghent CM, Howard CJ, Hartooni N, Safari M, Frankel AD, Morgan DO (2020). Phosphoregulation of phase separation by the SARS-CoV-2 N protein suggests a biophysical basis for its dual functions. Mol Cell.

[CR7] Chan JF, Kok KH, Zhu Z, Chu H, To KK, Yuan S, Yuen KY (2020). Genomic characterization of the 2019 novel human-pathogenic coronavirus isolated from a patient with atypical pneumonia after visiting Wuhan. Emerg Microbes Infect.

[CR8] Chen H, Cui Y, Han X, Hu W, Sun M, Zhang Y, Wang PH, Song G, Chen W, Lou J (2020). Liquid-liquid phase separation by SARS-CoV-2 nucleocapsid protein and RNA. Cell Res.

[CR9] Chen J, Fan J, Chen Z, Zhang M, Peng H, Liu J, Ding L, Liu M, Zhao C, Zhao P (2021). Nonmuscle myosin heavy chain IIA facilitates SARS-CoV-2 infection in human pulmonary cells. Proc Natl Acad Sci USA.

[CR10] Cohen ME, Eichel R, Steiner-Birmanns B, Janah A, Ioshpa M, Bar-Shalom R, Paul JJ, Gaber H, Skrahina V, Bornstein NM (2020). A case of probable Parkinson's disease after SARS-CoV-2 infection. Lancet Neurol.

[CR11] Conicella AE, Zerze GH, Mittal J, Fawzi NL (2016). ALS mutations disrupt phase separation mediated by alpha-helical structure in the TDP-43 low-complexity C-terminal domain. Structure.

[CR12] Crunfli F, Carregari VC, Veras FP, Vendramini PH, Fragnani Valença AG, Marcelo Antunes ASL, Brandão-Teles C, da Silva Zuccoli G, Reis-de-Oliveira G, Silva-Costa LC, et al (2021) SARS-CoV-2 infects brain astrocytes of COVID-19 patients and impairs neuronal viability. medRxiv

[CR13] Cubuk J, Alston JJ, Incicco JJ, Singh S, Stuchell-Brereton MD, Ward MD, Zimmerman MI, Vithani N, Griffith D, Wagoner JA, et al (2020) The SARS-CoV-2 nucleocapsid protein is dynamic, disordered, and phase separates with RNA. bioRxiv 10.1038/s41467-021-21953-3 PMC8007728 33782395

[CR14] De Santis R, Alfano V, de Turris V, Colantoni A, Santini L, Garone MG, Antonacci G, Peruzzi G, Sudria-Lopez E, Wyler E (2019). Mutant FUS and ELAVL4 (HuD) aberrant crosstalk in amyotrophic lateral sclerosis. Cell Rep.

[CR15] Dormann D, Rodde R, Edbauer D, Bentmann E, Fischer I, Hruscha A, Than ME, Mackenzie IR, Capell A, Schmid B (2010). ALS-associated fused in sarcoma (FUS) mutations disrupt transportin-mediated nuclear import. EMBO J.

[CR16] Duan Y, Du A, Gu J, Duan G, Wang C, Gui X, Ma Z, Qian B, Deng X, Zhang K (2019). PARylation regulates stress granule dynamics, phase separation, and neurotoxicity of disease-related RNA-binding proteins. Cell Res.

[CR17] Eimer WA, Vijaya Kumar DK, Navalpur Shanmugam NK, Rodriguez AS, Mitchell T, Washicosky KJ, Gyorgy B, Breakefield XO, Tanzi RE, Moir RD (2018). Alzheimer's disease-associated beta-amyloid is rapidly seeded by herpesviridae to protect against brain infection. Neuron.

[CR18] Gao C, Zeng J, Jia N, Stavenhagen K, Matsumoto Y, Zhang H, Li J, Hume AJ, Muhlberger E, van Die I, et al (2020) SARS-CoV-2 spike protein interacts with multiple innate immune receptors. bioRxiv

[CR19] Gatto EM, Fernandez Boccazzi J (2020). COVID-19 and neurodegeneration: what can we learn from the past?. Eur J Neurol.

[CR20] Gordon DE, Jang GM, Bouhaddou M, Xu J, Obernier K, White KM, O'Meara MJ, Rezelj VV, Guo JZ, Swaney DL (2020). A SARS-CoV-2 protein interaction map reveals targets for drug repurposing. Nature.

[CR21] Gu J, Wang C, Hu R, Li Y, Zhang S, Sun Y, Wang Q, Li D, Fang Y, Liu C (2021). Hsp70 chaperones TDP-43 in dynamic, liquid-like phase and prevents it from amyloid aggregation. Cell Res.

[CR22] Gu Y, Cao J, Zhang X, Gao H, Wang Y, Wang J, He J, Jiang X, Zhang J, Shen G, et al (2021b) Receptome profiling identifies KREMEN1 and ASGR1 as alternative functional receptors of SARS-CoV-2. Cell Res 10.1038/s41422-021-00595-6 PMC8617373 34837059

[CR23] Guseva S, Milles S, Jensen MR, Salvi N, Kleman JP, Maurin D, Ruigrok RWH, Blackledge M (2020). Measles virus nucleo- and phosphoproteins form liquid-like phase-separated compartments that promote nucleocapsid assembly. Sci Adv.

[CR24] Gwon Y, Maxwell BA, Kolaitis RM, Zhang P, Kim HJ, Taylor JP (2021). Ubiquitination of G3BP1 mediates stress granule disassembly in a context-specific manner. Science.

[CR25] Hascup ER, Hascup KN (2020). Does SARS-CoV-2 infection cause chronic neurological complications?. Geroscience.

[CR26] Heneka MT, Golenbock D, Latz E, Morgan D, Brown R (2020). Immediate and long-term consequences of COVID-19 infections for the development of neurological disease. Alzheimers Res Ther.

[CR27] Iserman C, Roden CA, Boerneke MA, Sealfon RSG, McLaughlin GA, Jungreis I, Fritch EJ, Hou YJ, Ekena J, Weidmann CA (2020). Genomic RNA elements drive phase separation of the SARS-CoV-2 nucleocapsid. Mol Cell.

[CR28] Jain S, Wheeler JR, Walters RW, Agrawal A, Barsic A, Parker R (2016). ATPase-modulated stress granules contain a diverse proteome and substructure. Cell.

[CR29] Jang H, Boltz D, Sturm-Ramirez K, Shepherd KR, Jiao Y, Webster R, Smeyne RJ (2009). Highly pathogenic H5N1 influenza virus can enter the central nervous system and induce neuroinflammation and neurodegeneration. Proc Natl Acad Sci USA.

[CR30] Johnson BS, Snead D, Lee JJ, McCaffery JM, Shorter J, Gitler AD (2009). TDP-43 is intrinsically aggregation-prone, and amyotrophic lateral sclerosis-linked mutations accelerate aggregation and increase toxicity. J Biol Chem.

[CR31] Kato M, Han TW, Xie S, Shi K, Du X, Wu LC, Mirzaei H, Goldsmith EJ, Longgood J, Pei J (2012). Cell-free formation of RNA granules: low complexity sequence domains form dynamic fibers within hydrogels. Cell.

[CR32] Kaur R, Lal SK (2020). The multifarious roles of heterogeneous ribonucleoprotein A1 in viral infections. Rev Med Virol.

[CR33] Kim HJ, Kim NC, Wang YD, Scarborough EA, Moore J, Diaz Z, MacLea KS, Freibaum B, Li S, Molliex A (2013). Mutations in prion-like domains in hnRNPA2B1 and hnRNPA1 cause multisystem proteinopathy and ALS. Nature.

[CR34] Klingstedt T, Shirani H, Aslund KO, Cairns NJ, Sigurdson CJ, Goedert M, Nilsson KP (2013). The structural basis for optimal performance of oligothiophene-based fluorescent amyloid ligands: conformational flexibility is essential for spectral assignment of a diversity of protein aggregates. Chemistry.

[CR35] Kwiatkowski TJ, Bosco DA, Leclerc AL, Tamrazian E, Vanderburg CR, Russ C, Davis A, Gilchrist J, Kasarskis EJ, Munsat T (2009). Mutations in the FUS/TLS gene on chromosome 16 cause familial amyotrophic lateral sclerosis. Science.

[CR36] Lai MM, Cavanagh D (1997). The molecular biology of coronaviruses. Adv Virus Res.

[CR37] Lee IC, Huo TI, Huang YH (2020). Gastrointestinal and liver manifestations in patients with COVID-19. J Chin Med Assoc.

[CR38] Li W, Moore MJ, Vasilieva N, Sui J, Wong SK, Berne MA, Somasundaran M, Sullivan JL, Luzuriaga K, Greenough TC (2003). Angiotensin-converting enzyme 2 is a functional receptor for the SARS coronavirus. Nature.

[CR39] Li H, Xue Q, Xu X (2020). Involvement of the nervous system in SARS-CoV-2 infection. Neurotox Res.

[CR40] Liu Z, Zhang S, Gu J, Tong Y, Li Y, Gui X, Long H, Wang C, Zhao C, Lu J (2020). Hsp27 chaperones FUS phase separation under the modulation of stress-induced phosphorylation. Nat Struct Mol Biol.

[CR41] Luo L, Li Z, Ma P, Zou Y, Li P, Liang A, Jin Z, Chi T, Huang C, Zhang Y, et al (2020). SARS-CoV-2 Nucleocapsid protein impairs SG assembly by partitioning into G3BP condensate. SSRN Electron J

[CR42] Luo L, Li Z, Zhao T, Ju X, Ma P, Jin B, Zhou Y, He S, Huang J, Xu X (2021). SARS-CoV-2 nucleocapsid protein phase separates with G3BPs to disassemble stress granules and facilitate viral production. Sci Bull.

[CR43] Luo L, Li Z, Zhao T, Ju X, Ma P, Jin B, Zhou Y, He S, Huang J, Xu X (2021). SARS-CoV-2 nucleocapsid protein phase separates with G3BPs to disassemble stress granules and facilitate viral production. Sci Bull.

[CR44] Mao L, Jin H, Wang M, Hu Y, Chen S, He Q, Chang J, Hong C, Zhou Y, Wang D (2020). Neurologic manifestations of hospitalized patients with coronavirus disease 2019 in Wuhan. China. JAMA Neurol.

[CR45] Marreiros R, Muller-Schiffmann A, Trossbach SV, Prikulis I, Hansch S, Weidtkamp-Peters S, Moreira AR, Sahu S, Soloviev I, Selvarajah S (2020). Disruption of cellular proteostasis by H1N1 influenza A virus causes alpha-synuclein aggregation. Proc Natl Acad Sci USA.

[CR46] Matschke J, Lutgehetmann M, Hagel C, Sperhake JP, Schroder AS, Edler C, Mushumba H, Fitzek A, Allweiss L, Dandri M (2020). Neuropathology of patients with COVID-19 in Germany: a post-mortem case series. Lancet Neurol.

[CR47] McBride R, van Zyl M, Fielding BC (2014). The coronavirus nucleocapsid is a multifunctional protein. Viruses.

[CR48] Molliex A, Temirov J, Lee J, Coughlin M, Kanagaraj AP, Kim HJ, Mittag T, Taylor JP (2015). Phase separation by low complexity domains promotes stress granule assembly and drives pathological fibrillization. Cell.

[CR49] Monette A, Niu M, Chen L, Rao S, Gorelick RJ, Mouland AJ (2020). Pan-retroviral nucleocapsid-mediated phase separation regulates genomic RNA positioning and trafficking. Cell Rep.

[CR50] Moosa MM, Banerjee PR (2020). Subversion of host stress granules by coronaviruses: potential roles of pi-rich disordered domains of viral nucleocapsids. J Med Virol.

[CR51] Onomoto K, Jogi M, Yoo JS, Narita R, Morimoto S, Takemura A, Sambhara S, Kawaguchi A, Osari S, Nagata K (2012). Critical role of an antiviral stress granule containing RIG-I and PKR in viral detection and innate immunity. PLoS ONE.

[CR52] Paniz-Mondolfi A, Bryce C, Grimes Z, Gordon RE, Reidy J, Lednicky J, Sordillo EM, Fowkes M (2020). Central nervous system involvement by severe acute respiratory syndrome coronavirus-2 (SARS-CoV-2). J Med Virol.

[CR53] Patel A, Lee HO, Jawerth L, Maharana S, Jahnel M, Hein MY, Stoynov S, Mahamid J, Saha S, Franzmann TM (2015). A liquid-to-solid phase transition of the ALS protein FUS accelerated by disease mutation. Cell.

[CR54] Peng TY, Lee KR, Tarn WY (2008). Phosphorylation of the arginine/serine dipeptide-rich motif of the severe acute respiratory syndrome coronavirus nucleocapsid protein modulates its multimerization, translation inhibitory activity and cellular localization. FEBS J.

[CR55] Perdikari TM, Murthy AC, Ryan VH, Watters S, Naik MT, Fawzi NL (2020). SARS-CoV-2 nucleocapsid protein phase-separates with RNA and with human hnRNPs. EMBO J.

[CR56] Protter DSW, Parker R (2016). Principles and properties of stress granules. Trends Cell Biol.

[CR57] Qamar S, Wang G, Randle SJ, Ruggeri FS, Varela JA, Lin JQ, Phillips EC, Miyashita A, Williams D, Strohl F (2018). FUS phase separation is modulated by a molecular chaperone and methylation of arginine cation-pi interactions. Cell.

[CR58] Readhead B, Haure-Mirande JV, Funk CC, Richards MA, Shannon P, Haroutunian V, Sano M, Liang WS, Beckmann ND, Price ND (2018). Multiscale analysis of independent Alzheimer's cohorts finds disruption of molecular, genetic, and clinical networks by human herpesvirus. Neuron.

[CR59] Saikatendu KS, Joseph JS, Subramanian V, Neuman BW, Buchmeier MJ, Stevens RC, Kuhn P (2007). Ribonucleocapsid formation of severe acute respiratory syndrome coronavirus through molecular action of the N-terminal domain of N protein. J Virol.

[CR60] Savastano A, Ibanez de Opakua A, Rankovic M, Zweckstetter M (2020). Nucleocapsid protein of SARS-CoV-2 phase separates into RNA-rich polymerase-containing condensates. Nat Commun.

[CR61] Serrano-Castro PJ, Estivill-Torrus G, Cabezudo-Garcia P, Reyes-Bueno JA, Ciano Petersen N, Aguilar-Castillo MJ, Suarez-Perez J, Jimenez-Hernandez MD, Moya-Molina MA, Oliver-Martos B (2020). Impact of SARS-CoV-2 infection on neurodegenerative and neuropsychiatric diseases: a delayed pandemic?. Neurologia.

[CR62] Singal CMS, Jaiswal P, Seth P (2020). SARS-CoV-2, more than a respiratory virus: its potential role in neuropathogenesis. ACS Chem Neurosci.

[CR63] Song E, Zhang C, Israelow B, Lu-Culligan A, Prado AV, Skriabine S, Lu P, Weizman OE, Liu F, Dai Y (2021). Neuroinvasion of SARS-CoV-2 in human and mouse brain. J Exp Med.

[CR64] Soresina A, Moratto D, Chiarini M, Paolillo C, Baresi G, Foca E, Bezzi M, Baronio B, Giacomelli M, Badolato R (2020). Two X-linked agammaglobulinemia patients develop pneumonia as COVID-19 manifestation but recover. Pediatr Allergy Immunol.

[CR65] Thevarajan I, Nguyen THO, Koutsakos M, Druce J, Caly L, van de Sandt CE, Jia X, Nicholson S, Catton M, Cowie B (2020). Breadth of concomitant immune responses prior to patient recovery: a case report of non-severe COVID-19. Nat Med.

[CR66] Wolozin B, Ivanov P (2019). Stress granules and neurodegeneration. Nat Rev Neurosci.

[CR67] Wu F, Zhao S, Yu B, Chen YM, Wang W, Song ZG, Hu Y, Tao ZW, Tian JH, Pei YY (2020). A new coronavirus associated with human respiratory disease in China. Nature.

[CR68] Zhang X, Wang F, Hu Y, Chen R, Meng D, Guo L, Lv H, Guan J, Jia Y (2020). In vivo stress granule misprocessing evidenced in a FUS knock-in ALS mouse model. Brain.

[CR69] Zhao D, Xu W, Zhang X, Wang X, Ge Y, Yuan E, Xiong Y, Wu S, Li S, Wu N (2021). Understanding the phase separation characteristics of nucleocapsid protein provides a new therapeutic opportunity against SARS-CoV-2. Protein Cell.

[CR70] Zhou P, Yang XL, Wang XG, Hu B, Zhang L, Zhang W, Si HR, Zhu Y, Li B, Huang CL (2020). A pneumonia outbreak associated with a new coronavirus of probable bat origin. Nature.

[CR71] Zhu N, Zhang D, Wang W, Li X, Yang B, Song J, Zhao X, Huang B, Shi W, Lu R (2020). A novel coronavirus from patients with pneumonia in China, 2019. N Engl J Med.

