# Supplementary material for: SARS-CoV-2 impairs the disassembly of stress granules and promotes ALS-associated amyloid aggregation
Source: Protein Cell. 2022 Apr 6;13(8):602–14. doi: 10.1007/s13238-022-00905-7 (PMC8983322; doi:10.1007/s13238-022-00905-7)
Supplement: Supplementary file 1 — Supplementary file1 (PDF 1645 kb) [file 13238_2022_905_MOESM1_ESM.pdf]

Supplementary information

SARS-CoV-2 impairs the disassembly of stress granules and promotes ALS-associated amyloid aggregation

Yichen Li[1,2#], Shuaiyao Lu[3,4#], Jinge Gu[6,7#], Wencheng Xia[6,7], Shengnan Zhang[6,7], Shenqing Zhang[1,2], Yan Wang[8], Chong Zhang[8], Yunpeng Sun[6,7], Jian Lei[8], Cong Liu[6,7], Zhaoming Su[8*], Juntao Yang[5*], Xiaozhong Peng[3,4*], Dan Li[1,9,10*]

[1]Bio-X Institutes, Key Laboratory for the Genetics of Developmental and Neuropsychiatric Disorders, Ministry of Education, Shanghai Jiao Tong University, Shanghai, 200030, China;

[2]School of Life Sciences and Biotechnology, Shanghai Jiao Tong University, Shanghai 200030, China；

[3]National Kunming High-level Biosafety Primate Research Center, Institute of Medical Biology, Chinese Academy of Medical Sciences and Peking Union Medical College, Yunnan, China;

[4]State Key Laboratory of Medical Molecular Biology, Chinese Academy of Medical Sciences, School of Basic Medicine, Peking Union Medical College, Beijing, China;

[5]State Key Laboratory of Medical Molecular Biology, Department of Biochemistry and Molecular Biology, Institute of Basic Medical Sciences, Chinese Academy of Medical Sciences & Peking Union Medical College, Beijing, China.

[6]Interdisciplinary Research Center on Biology and Chemistry, Shanghai Institute of Organic Chemistry, Chinese Academy of Sciences, Shanghai 201210, China;

[7]University of Chinese Academy of Sciences, Beijing 100049, China;

[8]State Key Laboratory of Biotherapy and Cancer Center, National Clinical Research Center for Geriatrics, West China Hospital, Sichuan University, Sichuan 610041, China;

[9]Bio-X-Renji Hospital Research Center, Renji Hospital, School of Medicine, Shanghai Jiao Tong University, Shanghai, 200240, China;

[10]Zhangjiang Institute for Advanced Study, Shanghai Jiao Tong University, Shanghai 200240, China.

#These authors contributed equally to this work.

*Correspondence: D. L. (lidan2017@sjtu.edu.cn); X. P. (pengxiaozhong@pumc.edu.cn); J. Y. (yangjt@pumc.edu.cn); Z. S. (zsu@scu.edu.cn).

Material and methods

Plasmid construction, protein expression and purification:

For *E. coli* expression, full-length SARS-CoV-2 nucleocapsid N protein was cloned into vector pET28a with an N-terminal His_6 tag. Full-length FUS and TDP43 were cloned into pET32a vector containing an MBP-His_6 tag fused at the N terminus with a HRV 3C protease cleavage site followed by an EGFP tag at the C terminus. Full-length hnRNPA1 was cloned into pET9d vector. HnRNPA1-LC (residues 186-320) was cloned into pET32a vector with a Trx1- His_6 tag at the N terminus. TDP43-LC (residues 274-414) and FUS-LC (residues 1-163) were cloned into pET28a and pET22b vectors, respectively, with an N-terminal His_6 tag. For protein overexpression in mammalian cell lines, N protein and ACE2 were subcloned into pcDNA3.1 vector fused with an N-terminal flag tag. CFP-FUS-P525L and RFP-N were subcloned into pCAG vector. mEGFP-G3BP1 was subcloned into pCMV vector.

N protein construct was induced to express in *E. coli* BL21 (DE3) after adding 1 mM IPTG. After induction, cells grew overnight at 16 ℃, then the cells were harvested and lysed in lysis buffer containing 50 mM Tris-HCl (pH 7.5), 500 mM NaCl, 12.5 mM imidazole, 4 mM β-mercaptoethanol, 2 mM PMSF and 100 $\mu g \cdot ml^{-1}$ RNase A (Roche, 10109142001). The cellular lysate was centrifugated to remove the precipitates and subjected to Ni column (GE Healthcare). The protein was eluted with 25 mM Tris-HCl (pH 7.5), 100 mM NaCl, and 250 mM imidazole and then concentrated and subjected to size-exclusion chromatography column Superdex 200 16/600 (GE Healthcare) in 25 mM Tris-HCl (pH 7.5), 100 mM NaCl and 2 mM DTT.

Full-length MBP-FUS-EGFP was expressed in *E. coli* BL21 (DE3) cells overnight at 16 ℃ after incubation with 0.5 mM IPTG. Cells were lysed and subjected to Ni column in 50 mM Tris-HCl (pH 8.0), 500 mM NaCl, 10 mM imidazole, 4 mM β-mercaptoethanol, and 100 $\mu g \cdot ml^{-1}$ RNase A, and 1 mM PMSF. The protein was eluted with 50 mM Tris-HCl (pH 8.0), 500 mM NaCl, 100 mM imidazole, and 4 mM β-mercaptoethanol. Then the MBP-His_6 tag was cleaved by GST-tagged 3C prescission protease during dialysis in 50 mM Tris-HCl (pH 7.4), 1 M

KCl, 10% glycerol, 4 mM β-mercaptoethanol for 4 h. The cleaved MBP-His and 3C protease were further removed with Ni column and Glutathione Sepharose column (GE Healthcare). Then the protein was concentrated and subjected into size-exclusion chromatography column Superdex 200 16/600 in 50 mM Tris-HCl (pH 7.5), 500 mM KCl, 2 mM DTT and 10% glycerol.

Full-length MBP-TDP43-EGFP was expressed in *E. coli* BL21 (DE3) cells overnight at 16 ℃ after incubation at an optical density of 0.8 ($OD_{600\ nm}$) with 0.5 mM IPTG. Cells were lysed and subjected to Ni column in 50 mM Tris (pH 7.5), 500 mM NaCl, 2 mM β-mercaptoethanol, and 100 $\mu g \cdot ml^{-1}$ RNase A, and 1 mM PMSF. The protein was eluted with 50 mM Tris-HCl (pH 7.5), 500 mM NaCl, 100 mM imidazole, 2 mM β-mercaptoethanol. And then the protein was concentrated and subjected to size-exclusion chromatography column Superdex 200 16/600 in 50 mM Tris-HCl (pH 7.5), 500 mM NaCl, 2 mM DTT, and 5% glycerol.

Full-length hnRNPA1 was expressed in *E. coli* BL21 (DE3) pLysS overnight at 25℃ after incubation with 0.4 mM IPTG. Cells were lysed and subjected to SP FF column (GE Healthcare) in 50 mM Tris-HCl (pH 7.5), 100 $\mu g \cdot ml^{-1}$ RNase A, 2 mM DTT, and 1 mM PMSF. The protein was eluted with 50 mM Tris-HCl (pH 7.5), 300 mM NaCl, 2 mM DTT and then concentrated, and further subjected to size-exclusion chromatography column Superdex 75 16/60 (GE Healthcare) in 50 mM Tris-HCl (pH 7.5), 500 mM NaCl, and 2 mM DTT.

FUS-LC (residues 1-163) was expressed in *E. coli* BL21 (DE3) which was induced with 0.5 mM IPTG at 16 ℃ for 48 h. Cells were lysed by sonication in 50 mM Tris-HCl (pH 8.0), 6 M guanidine hydrochloride on the ice. Cell supernatant was loaded onto a Ni column. Protein was eluted in 50 mM Tris-HCl (pH 8.0), 6 M guanidine hydrochloride and 50 mM imidazole. The eluted protein was purified via high performance liquid chromatography (HPLC) (Agilent) and freeze dried by FreeZone lyophilizer (Thermo Fisher). FUS-LC powder was dissolved into 50 mM Tris-HCl (pH 8.0), 8 M urea and then desalted into 5 mM CAPS (pH 11.0), and the concentrated FUS-LC was diluted into each individual buffer solution for further experiments.

HnRNPA1-LC with an N-terminal Trx1-His_6 tag (residues 186-320) was expressed in *E. coli* BL21 (DE3) at 25 ℃ overnight after induction by 0.4 mM IPTG. The protein was purified with Ni column and HPLC similar to the FUS-LC purification procedure. Trx1-His_6-hnRNPA1-LC powder was dissolved into 50 mM Tris-HCl (pH 7.5) and 100 mM NaCl. Then, the Trx1 tag was cleaved by 3C protease with a 50:1 (protein: protease) molar ratio at room temperature overnight. The hnRNPA1 LC protein without tag was obtained via HPLC purification followed by lyophilization. .

TDP43-LC (residues 274-414) was expressed in *E. coli* BL21 (DE3) with 1 mM IPTG at 37 ℃ for 10 h. Cells were lysed by sonication in 50 mM Tris-HCl (pH 8.0) and 6 M guanidine hydrochloride on the ice. Cell supernatant was loaded onto a Ni column. Protein was eluted in 50 mM Tris-HCl (pH 8.0), 6 M guanidine hydrochloride and 50 mM imidazole. The protein was further desalted into 50 mM Tris-HCl (pH 8.0), 8 M urea. The concentrated TDP43-LC was aliquoted in PCR tubes, flash-frozen in liquid nitrogen and stored at -80 ℃. The frozen protein was then desalted into different buffers for further experiments.

Cell culture and transfection

HeLa cells (Cell Bank of the Chinese Academy of Sciences, Shanghai, SCSP-504) were cultured in the Dulbecco's modified Eagle's medium (sigma, D0819) supplemented with 10% fetal bovine serum (FBS, BioWest), 1% penicillin/streptomcyin. Vero cells were cultured in high-glucose DMEM (Gibco, Cat# 11995500BT) supplemented with 10% fetal bovine serum (FBS, Gibco, Cat# 10099-141C) and 1% penicillin/streptomycin. All cells were incubated at 37 ℃ in a humidified atmosphere of 95% air and 5% CO_2. PolyJet™ reagent (SignaGen, SL100688) was used for the transfection of plasmids into HeLa cells. Cells were transfected for 24 h before proceeding with subsequent experiments.

Virus amplification and infection

SARS-CoV-2 viral stock was kindly provided by the Center of Diseases Control, Guangdong Province China. Viruses were amplified in Vero cells and then confirmed via RT-PCR, sequencing and TEM, and titrated via plaque assay. HeLa cells or Vero cells were grown on coverslips in the 24-well plate, and HeLa cells were infected with 100 μl SARS-CoV-2 at a MOI of 0.75. HeLa cells transiently expressing human ACE2 were infected with 100 μl SARS-CoV-2 at a MOI of 0.1. Vero cells were infected with 100 μL SARS-CoV-2 at a MOI of 0.05, and then cells were incubated at 37 ℃ in a humidified atmosphere of 95% air and 5% CO_2 for 0.5 h. Then the medium containing SARS-CoV-2 was removed and the cells were incubated in the fresh medium with 2% fetal bovine serum (FBS, BioWest) for 48 h (ACE2-HeLa cells and Vero cells) or 72 h (HeLa cells). Cells were then treated with 100 μM of sodium arsenite for 1 h to induce SGs prior to fixation with 4% paraformaldehyde. Virus-inoculated plates were placed in clean bench for 48 h and then exposed to ultraviolet radiation for 1 h before the subsequent immunofluorescence assay. All procedures associated with virus infection assay were performed in the BSL-4 lab.

Immunocytochemistry and confocal imaging

HeLa cells and Vero cells were grown on coverslips in the 24-well plate and then fixed with 4% paraformaldehyde in PBS for 15 min at room temperature. Fixed cells were permeabilized for 10 min in 0.5% Triton X-100, then blocked with 3% goat serum in PBST (PBS + 0.1% Triton X-100) for 1h. Cells were incubated with anti-G3BP1 antibody (BD, 611127), anti-flag antibody (CST, 2368S) overnight at 4 ℃. Virus-infection cells were incubated with anti-G3BP1 antibody (BD, 611127), anti-Nucleocapsid antibody (GeneTex, GTX135357), anti-Spike antibody (GeneTex, GTX632604), anti-Nsp1 antibody (GeneTex, GTX135612), anti-Nsp8 antibody (GeneTex, GTX632696), anti-ORF7α antibody (GeneTex, GTX632602) overnight at 4 ℃. AlexaFluor-488 (Invitrogen, A11001) and AlexaFluor-647 (Invitrogen, A11011) were used as the second antibodies, respectively. After 3 times washes with PBST, cells were mounted on glass slides using the ProLong™ Gold Antifade Mountant with DAPI (ThermoFisher, P36935). The samples were visualized by using the Leica TCS

SP8 confocal microscopy system using a 100× objective (oil immersion). Images were processed and assembled into figures using LAS X (Leica) and Fiji.

Assembly and disassembly of stress granules in cells

In virus-infected assay, HeLa cells were grown on coverslips in the 24-well plate, and HeLa cells were infected with 100 μl SARS-CoV-2 at a MOI of 0.75 for 72 h. HeLa cells transiently expressing human ACE2 were infected with 100 μl SARS-CoV-2 at a MOI of 0.1 for 48 h, and then were treated with 100 μM of sodium arsenite for 1 h to induce SGs after viral infection. For prolonged stress assay, ACE2-HeLa cells were infected with SARS-CoV-2 for 48 h, and then were treated with 100 μM of sodium arsenite for 1 h or 5 h to induce SGs after viral infection. For SGs disassembly, the medium containing sodium arsenite was removed and the cells were incubated in the fresh medium for the indicated time prior to fixation with 4% paraformaldehyde. Then cells were permeabilized and immunostained with anti-flag, anti-Nucleocapsid antibody (GeneTex, GTX135357) and anti-G3BP1 antibody (BD, 611127). As for the HeLa cells transfected with pcDNA3.1-flag-N or infected with SARS-CoV-2, the area of N protein containing SGs per cell was calculated using Fiji. All of the transfected or infected cells in the selected area were counted. In each experiment, at least 50 cells per slide were counted, data are shown as mean ± S.D., with n=3 biologically independent samples.

As for quantification of aggregated CFP-FUS with N protein, HeLa cells were grown on the coverslips in the 24-well plate, and were transfected with CFP-FUS P525L and Flag-N. Then the cells were fixed and immunostained with anti-flag antibody (CST, 2368S) after 24h transfection. Cells were washed with PBS three times and incubated with 20 μM pFTAA for 30 min, a high-affinity β-sheet aggregate chemical probe [1, 2]. In virus-infected assay, HeLa cells overexpressing ACE2 and CFP-FUS P525L were grown on the coverslips in the 24-well plate. Then the cells were infected with SARS-CoV-2 for 48h, and then fixed and immunostained with anti-Nucleocapsid antibody (GeneTex, GTX135357). The area of N-positive CFP-FUS aggregation per cell was calculated. All transfected cells in the selected

area were counted. In each experiment, at least 50 cells per slide were counted, data are shown as mean ± S.D., with n=3 biologically independent samples.

In vitro LLPS assay

In vitro LLPS experiments were performed at room temperature. The LLPS of N protein was induced by the addition of indicated concentrations of polyU (Sigma P9528) in LLPS buffer containing 25 mM Tris-HCl (pH 7.5), 100 mM NaCl and 2 mM DTT. For the co-LLPS, N protein with hnRNPA1-FL, MBP-TDP43-EGFP were incubated in a co-LLPS buffer (50 mM Tris-HCl, 100 mM NaCl, pH 7.5, 10% PEG 3,350). N protein was incubated with FUS-EGFP in the buffer containing 50 mM Tris-HCl (pH 7.5), 100 mM NaCl. All imaged were captured within 5 min after LLPS induction. Finally, 3 μL of each sample was pipetted onto a coverslip and imaged using a Leica microscope.

Protein fluorescent labeling

N protein was desalted into 50 mM NaPhosphate (pH 7.0), 50 mM NaCl. 10-fold (10:1) Oregon-Green488 (Invitrogen, O6149) or QSY7 (Invitrogen, Q10193) was incubated with N protein at 25 ℃ for 1 h, respectively. Then, the labeled proteins were further purified with a Superdex 75 10/300 size-exclusion chromatography column (GE Healthcare) in 50 mM NaPhosphate (pH 7.0), 50 mM NaCl. HnRNPA1 was desalted into 50 mM PBS (pH 7.0), 500 mM NaCl, and then 10-fold (10:1) Oregon-Green488 (Invitrogen, O6149) was incubated with hnRNPA1 at 4 ℃ for 12 h. The mixture was subjected to size-exclusion chromatography column Superdex 75 10/300 in 50 mM PBS (pH 7.0), 500 mM NaCl. The unlabeled protein was mixed with the fluorescent labeled protein with the molar ratio of 50:1 (unlabeled: labeled) for the following LLPS assay and confocal imaging.

Differential interference contrast (DIC) and fluorescent imaging for protein LLPS

For imaging of protein liquid droplets, the samples were dropped onto glass slide and sealed with coverslip. DIC images of N protein were acquired on a Leica TCS SP8 microscope with a 100× objective (oil immersion) at a resolution 2,048 × 2,048 pixel. Fluorescent images of the N protein phase-separated samples and co-LLPS samples between N protein and different SG proteins (FUS, hnRNPA1, TDP43) were acquired with the Leica TCS SP8 confocal microscopy using a 100× objective (oil immersion) at a resolution 2,048 × 2,048 pixel.

FUS phase transition and maturation assay

The samples contained 10 μM full-length FUS-EGFP in the presence or absence of 10 μM N protein in 50 mM Tris-HCl (pH 7.5), 100 mM NaCl. 20 μL samples were placed on the glass bottom of cell culture dish (40 mm×40 mm, 0.2 mm at thinnest bottom) (Nest, 80100) with coverslip. Leica TCS SP8 confocal microscopy was used to record images and fluorescence recovery after photobleaching (FRAP) data at indicated time points by using a 100× objective (oil immersion) with Z-stacks over 3 μm.

Fluorescence recovery after photobleaching (FRAP)

All FRAP experiments were performed on FRAP module of the Leica TCS SP8 confocal microscopy with a 100×objective (oil immersion). For *in vitro* FRAP assay, the time-lapse images were acquired at 488 nm. Acquisition was performed with 1,024 × 1,024 pixel. FRAP was restricted to a region of interest, defined graphically. A circular region was bleached for 2.58 s with full laser power, Post-bleach images were acquired at 2.58 s per frame. For each time point, the intensity was corrected by the intensity of a neighboring unbleached region.

For FRAP in living cells, HeLa cells were grown in chambered coverglass (Thermo Fisher Scientific, 155383), and then treated with 100 μM of sodium arsenite for 2 h to induce stress granules (SGs). SGs were bleached by using a 100% power 488 nm laser, Post-bleach images were acquired at 2.58 s per frame. Images were processes in Fiji and analyzed with the Leica Application Suite X software.

Nuclear magnetic resonance (NMR)

NMR experiments were performed at 298K on a Bruker 900 MHz spectrometer equipped with cryogenically cooled probes. ^{15}N-FUS-LC (25 μM) was prepared in NMR buffer containing 25 mM MES (pH 6.6), 150 mM NaCl, 10% glycerol, and 10% D_2O. ^{15}N-TDP43-LC (20 μM) was prepared in NMR buffer containing 20 mM MES (pH 6.0), 150 mM NaCl, 10% glycerol, and 20% D_2O. For titration experiments, each sample was prepared in a volume of 500 μL containing ^{15}N-FUS-LC (25 μM)/ ^{15}N-TDP43-LC (20 μM) and indicated concentrations of N protein. Backbone assignment of FUS-LC and TDP43-LC was accomplished based on previous studies [3, 4]. All NMR data were processed by NMRpipe and analyzed by SPARK [5, 6].

Thioflavin T (ThT) fluorescence assay

For ThT assay, 25 μM FUS-LC in buffer containing 50 mM Tris-HCl (pH 7.5), 100 mM NaCl, 50 μM ThT and 0.05% NaN_3 was incubated with 0.5% preformed FUS-LC fibril seeds (monomer equivalence) in 384-well plates (Corning), with indicated concentration of N protein. 25 μM TDP43-LC in buffer containing 50 mM Tris-HCl (pH 6.5), 100 mM NaCl, 50 μM ThT and 0.05% NaN_3 was incubated with indicated concentration of N protein in 384-well plates (Corning). 50 μM hnRNPA1-LC in buffer containing 50 mM Tris-HCl (pH 7.5), 100 mM NaCl, 50 μM ThT and 0.05% NaN_3 was incubated with indicated concentration of N protein in 384-well plates (Corning). ThT fluorescence was monitored using a Varioskan Flash spectral scanning multimode reader (Thermo Fisher Scientific) with excitation at 440 nm and emission at 485 nm at 25 ℃. The plate was shaken at 600 rpm for 10 seconds before each measurement of ThT fluorescent intensity. Four replicates were performed for each sample.

Negative-staining transmission electron microscopy (TEM)

5 μL of each sample was transferred to carbon-coated grids for 1 min and stained with 8 μL uranyl acetate (2%, v/v) for 45 s. Removing and drying the excess buffer on grids before acquiring TEM images. Grids were further assessed by using Tecnai G2 Spirit transmission electron microscope with 120 kV voltage.

Quantification and statistical analysis

All experiments were repeated, statistical parameters including the definitions and exact values of n (number of biological repeats), distributions and deviations are reported in the corresponding Figures and Figure Legends. Microsoft Excel or GraphPad Prism software were used for statistical analysis. All statistic values were displayed as mean ± S.D.. The statistical significance in this study is determined by the unpaired, two-tailed Student's *t*-test.

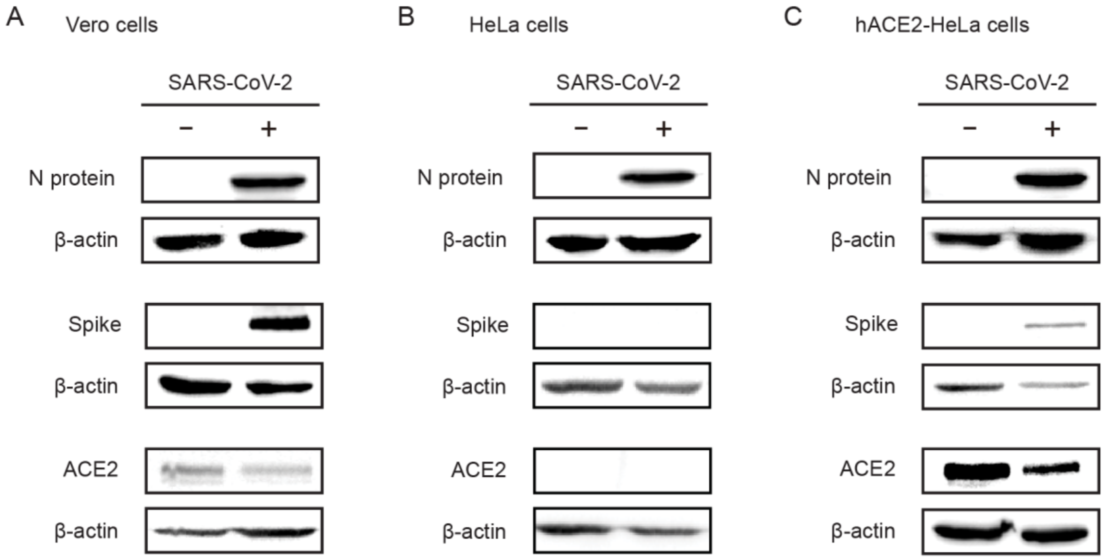

Supplementary Figure 1 Western blot analysis of the expression of SARS-CoV-2 proteins and ACE2 receptor in mammalian cells.

a, Monkey Vero cells. **b,** Human HeLa cells. **c,** HeLa cells transfected with human ACE2.

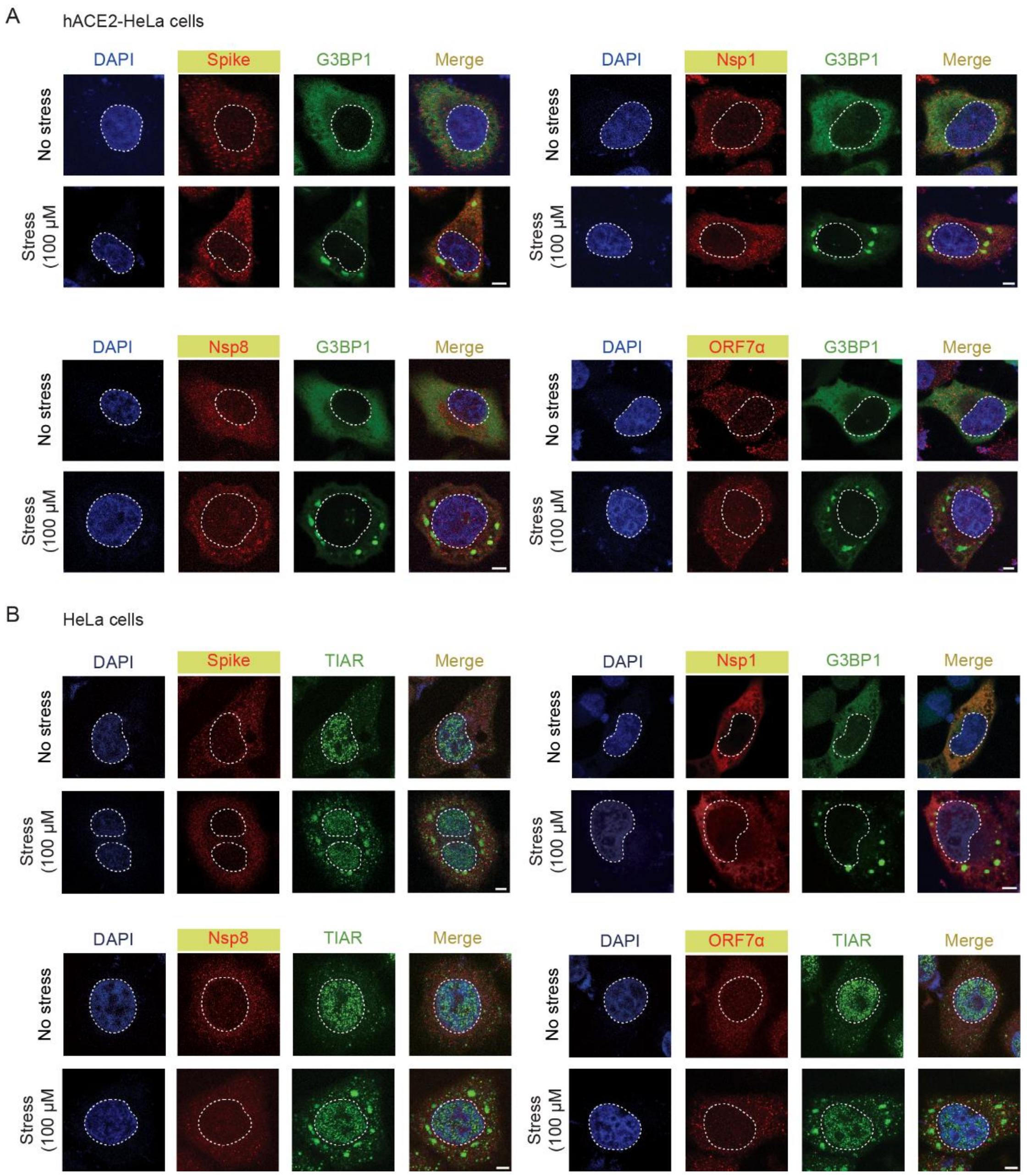

Supplementary Figure 2 Confocal images of viral proteins in ACE2-HeLa cells (a) and HeLa cells (b) infected by SARS-CoV-2.

Infected cells were stressed with 100 μM sodium arsenite for 1 h, and stained with DAPI, antibodies for the virus proteins and for SG marker proteins G3BP1 or TIAR. Arrows indicate SGs. Scale bar, 5 μm.

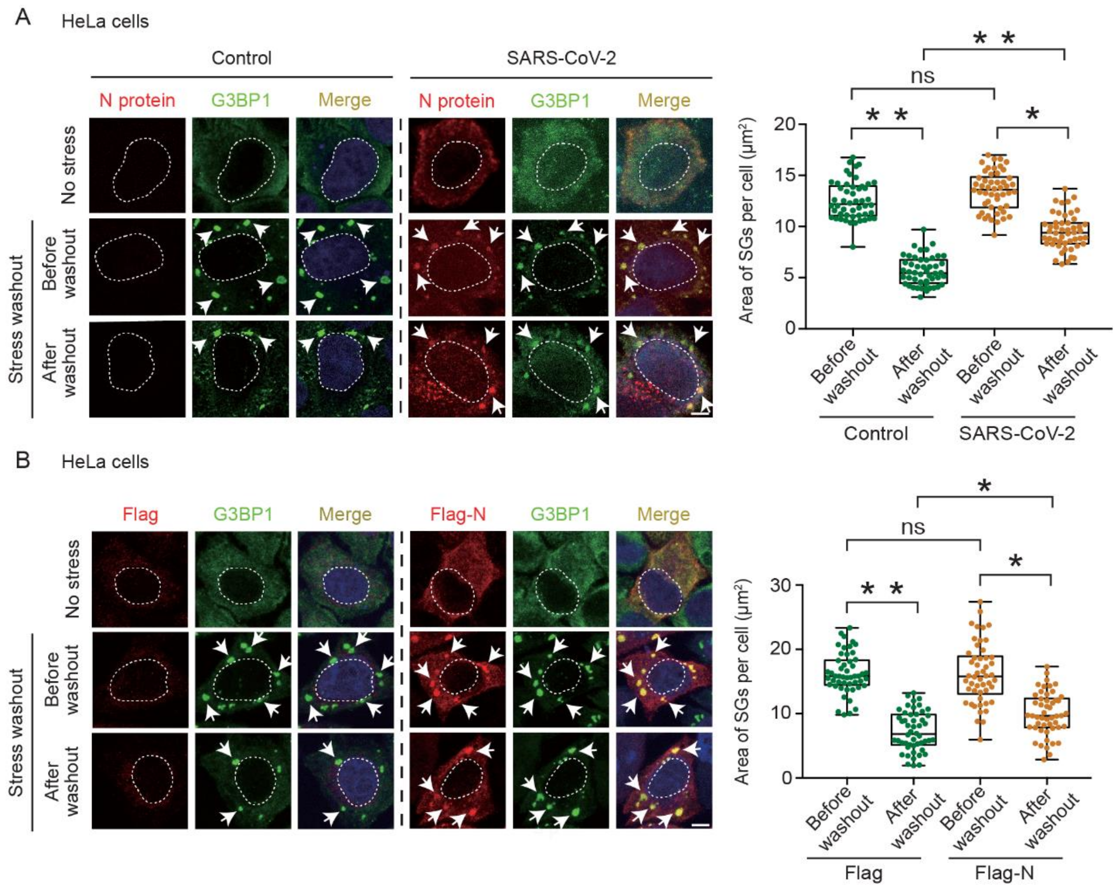

Supplementary Figure 3 SG self-disassembly in HeLa cells influence by N protein.

Confocal images of HeLa cells with or without (control) infection of SARS-CoV-2 (**a**) and HeLa cells overexpressing Flag-tagged N protein (**b**). Cells were stressed with 100 μM sodium arsenite for 1 h, followed by washing out sodium arsenite. Cells were stained with antibodies for viral N protein and SG marker protein G3BP1. Arrows indicate SGs. Scale bar, 5 μm. Quantitative analysis of the images is shown on the right as the area of SGs per cell. Values are means ±S.D., n > 150 cells from 3 replicates. Student's *t*-test, * $p<0.05$, ** $p<0.01$; ns, not significant.

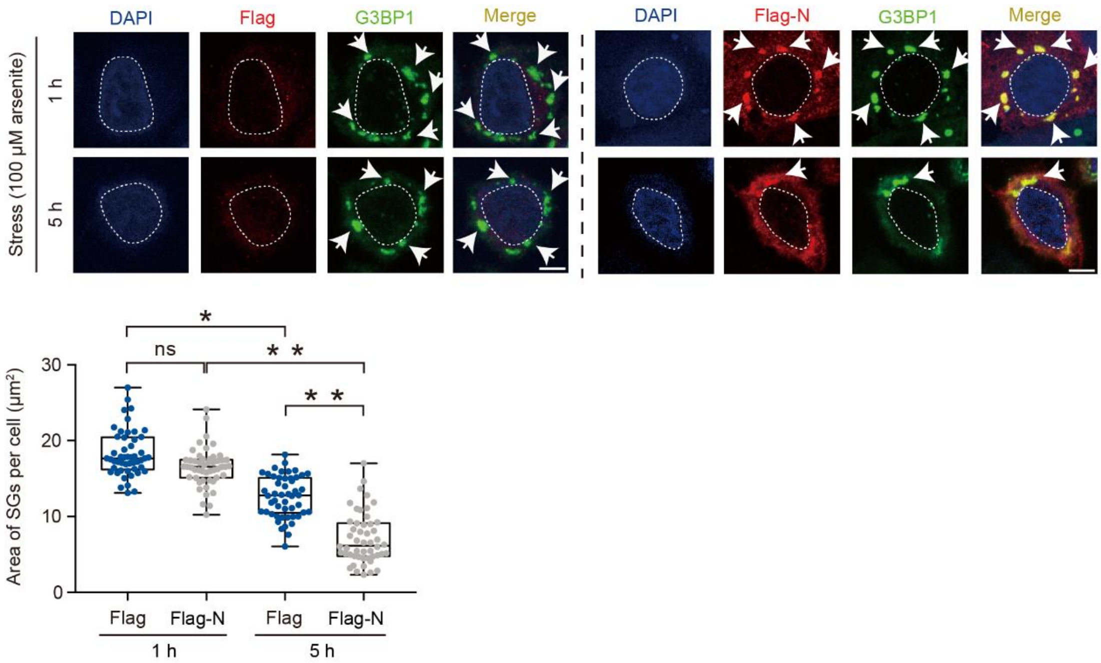

Supplementary Figure 4 SARS-CoV-2 N protein promotes the clearance of SGs in HeLa cells upon prolonged stress.

Confocal images of HeLa cells transfected with Flag (left) or Flag-N (right) are shown on top. Cells were stressed with 100 μM sodium arsenite for 1 h or 5 h. Cells were stained with DAPI, anti-flag, and anti-G3BP1. Arrows indicate stress granules. Scale bar, 5 μm. Quantitative analysis at the bottom is shown as the area of SGs per transfected cell. Values are means ±S.D., n > 150 cells from 3 replicates. Student's *t*-test, * $p<0.05$, ** $p<0.01$; ns, not significant.

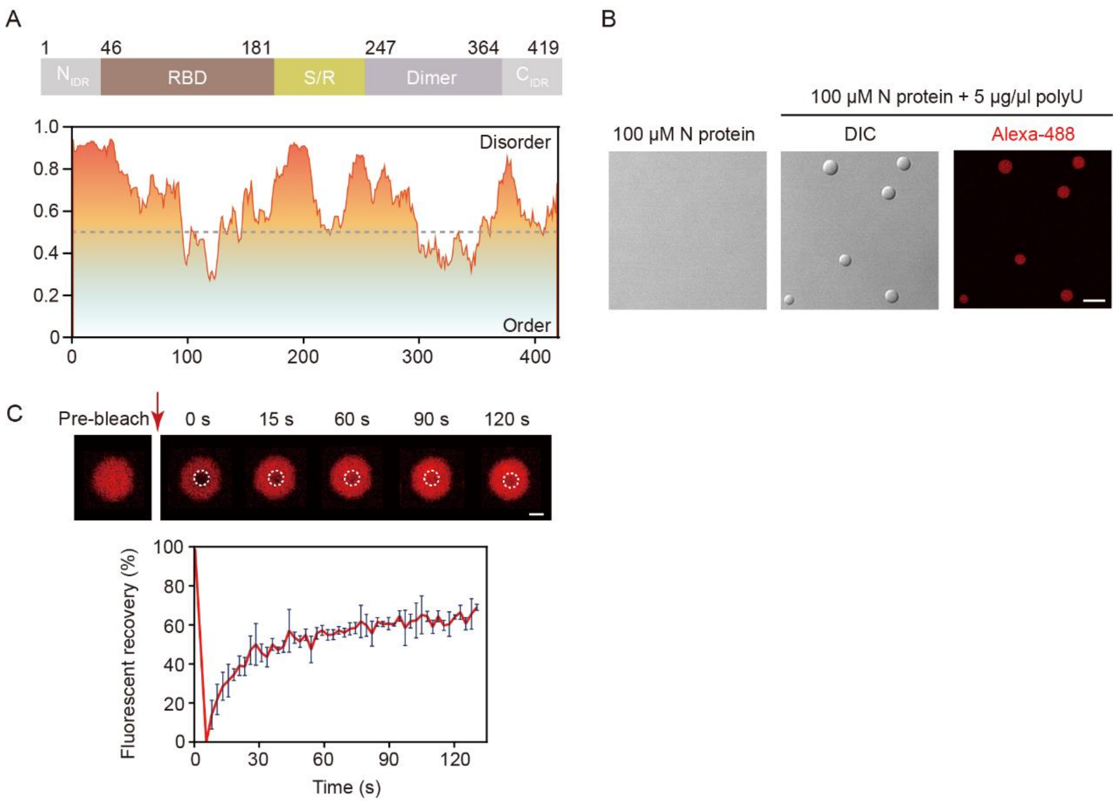

Supplementary Figure 5 Liquid-liquid phase separation of SARS-CoV-2 N protein.

a, Domain organization of SARS-CoV-2 N protein (top) and IDR prediction of N protein by IUPred [7] (bottom). **b,** Microscopic images of N protein droplets in the presence of synthetic single-stranded RNA (polyU) in the buffer containing 25 mM Tris-HCl, pH 7.5 and 100 mM NaCl. DIC: differential interference contrast microscopy. Alexa-488 is a chemical fluorescence label to mark N protein. Scale bar, 5 µm. **c,** FRAP montages of an N protein droplet formed in (**b**). The arrow indicates the action of bleaching. The graph (bottom) shows the recovery fraction as the function of time. Scale bar, 2 µm. Data shown are means ± S.D., n=3 individual droplets.

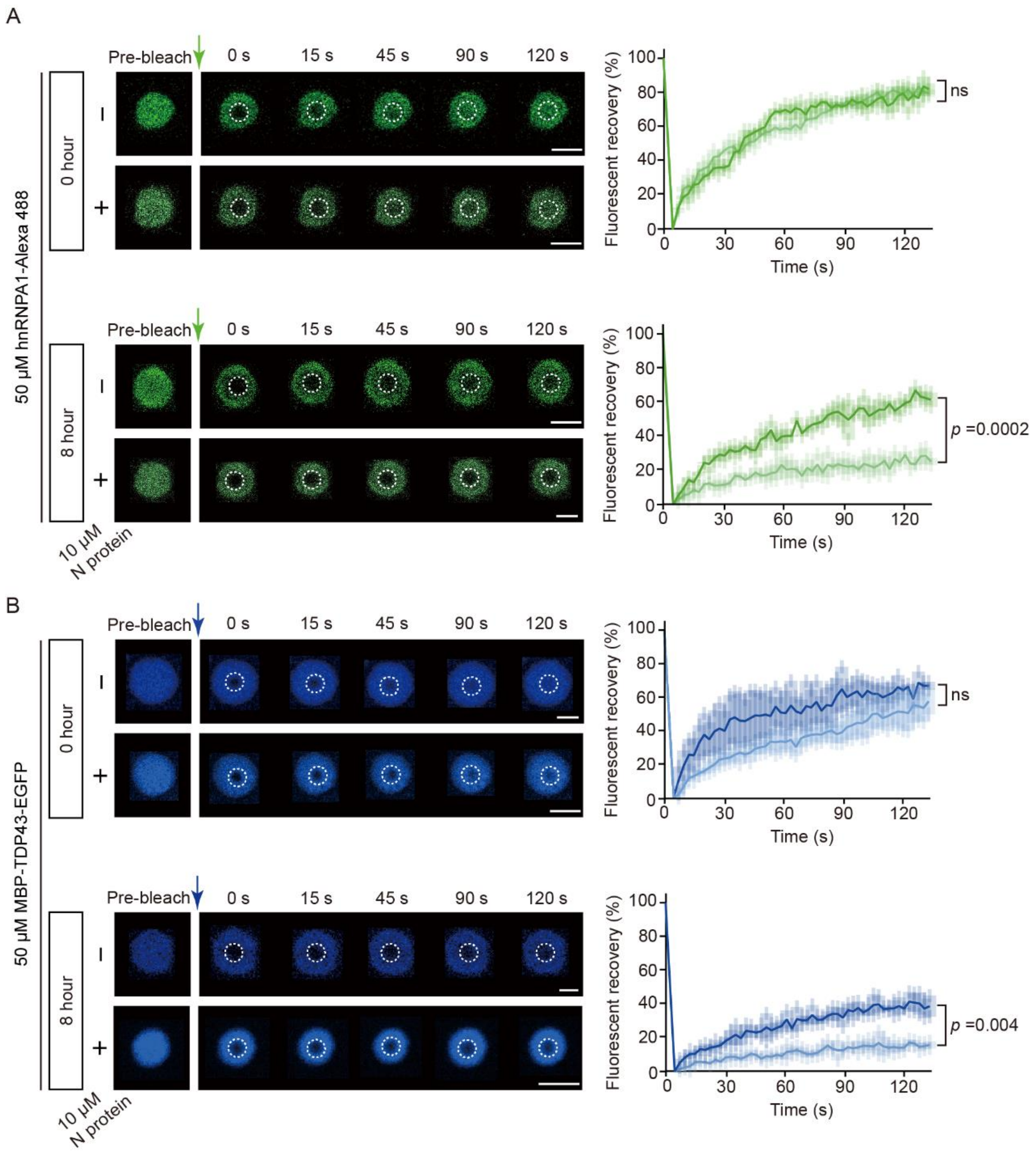

Supplementary Figure 6 SARS-CoV-2 N protein solidifies the liquid-like droplets of hnRNPA1 and TDP43.

FRAP montages of hnRNPA1 (**a**) and MBP-TDP43-EGFP (**b**) droplets are shown on the left. The arrows indicate the action of bleaching. Protein concentrations are indicated. Buffer: 50 mM Tris-HCl, pH 7.5, 100 mM NaCl, 10% PEG 3,350. The droplets are incubated for 0 h and 8 h, respectively. The graphs (right) show the recovery fraction as the function of time. Scale bar: 2 μm. Data shown are means ± S.D., n=3. Student's *t*-test. ns, not significant.

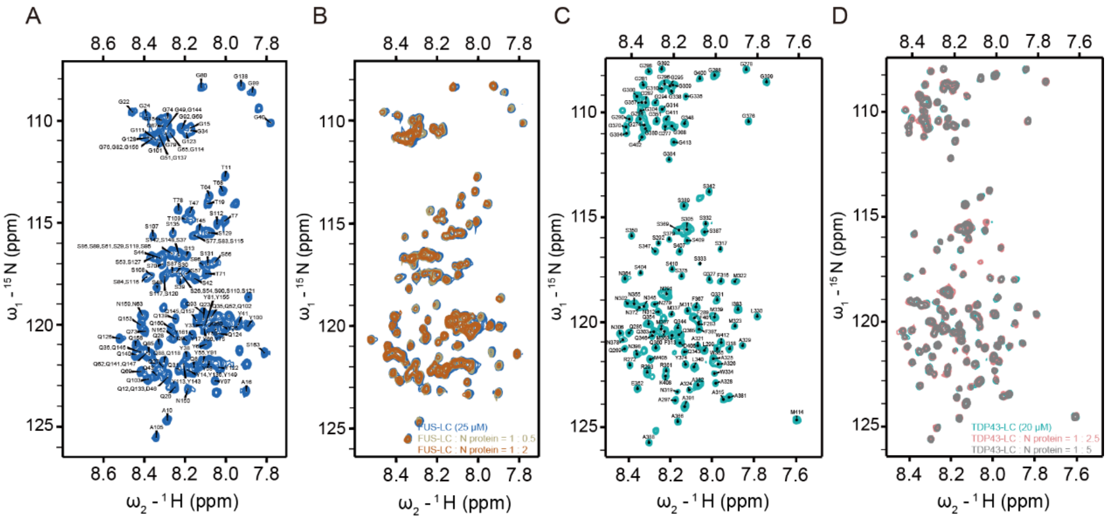

Supplementary Figure 7 NMR spectra of FUS-LC and TDP43-LC titrated with SARS-CoV-2 N protein.

a, Backbone assignment of FUS-LC (25 μM) with resonances labeled by one amino acid letter and the residue number in full-length FUS. **b,** Overlay of the 2D ^{1}H-^{15}N HSQC spectra of 25 μM ^{15}N-FUS-LC alone (blue) and in the presence of N protein at molar ratios (FUS-LC: N) of 1:0.5 (green) and 1:2 (orange), respectively. **c,** Backbone assignment of TDP43-LC (20 μM) with resonances labeled by one amino acid letter and the residue number. **d,** Overlay of the 2D ^{1}H-^{15}N HSQC spectra of 20 μM ^{15}N-TDP43-LC alone (cyan) and in the presence of N protein at molar ratios (TDP43-LC : N) of 1:2.5 (pink) and 1:5 (grey), respectively.

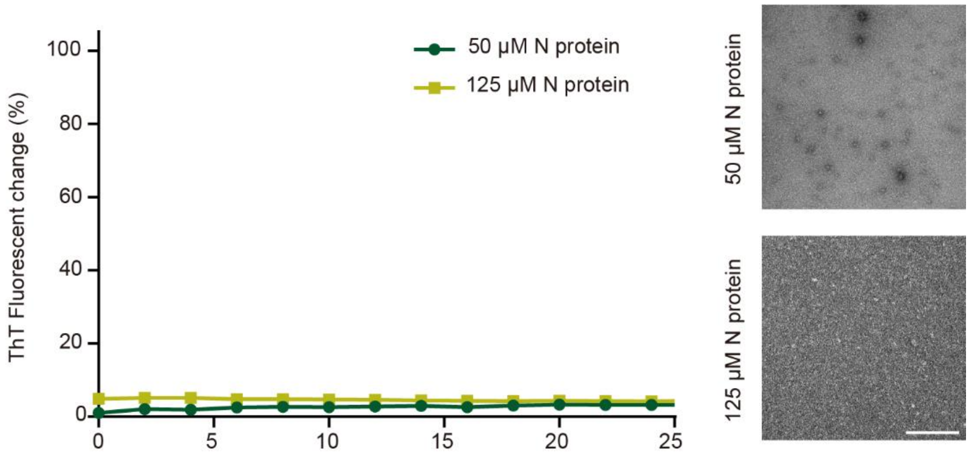

Supplementary Figure 8 ThT kinetic assay for SARS-CoV-2 N protein.
Protein concentrations are indicated. Buffer: 50 mM Tris-HCl, pH 7.5, 100 mM NaCl. Data correspond to mean ±S.D., n=3 independent samples. Scale bar, 500 nm.

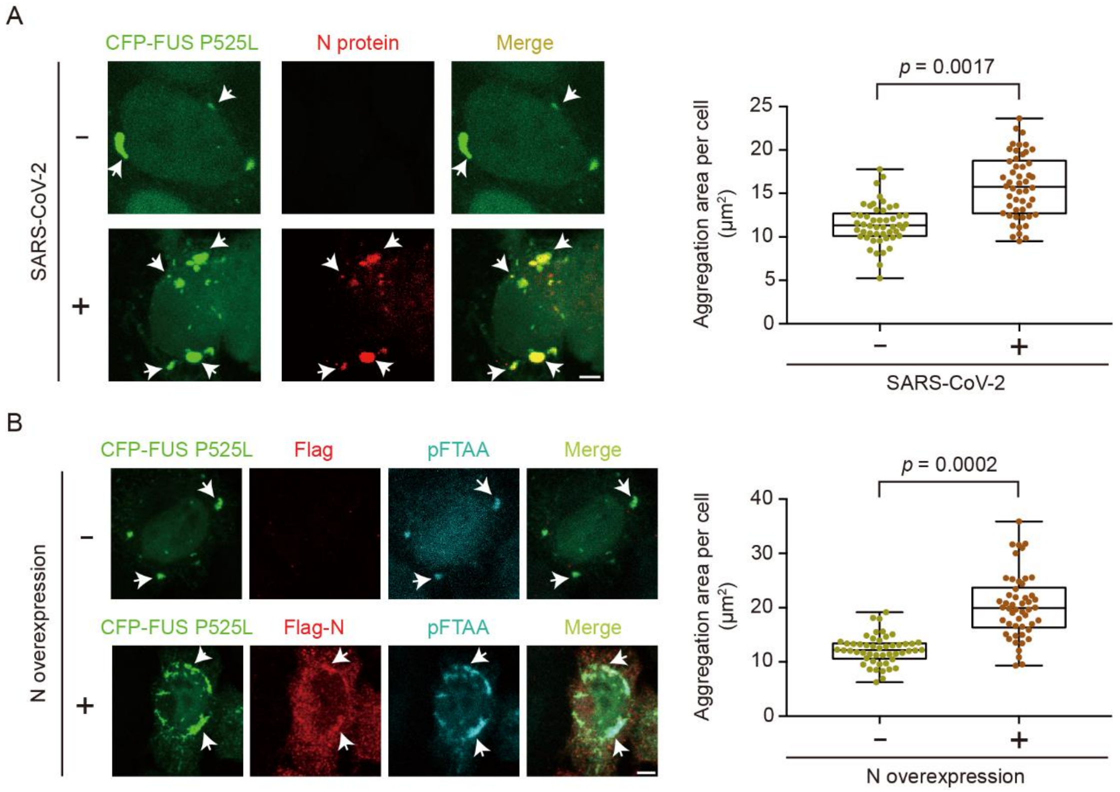

Supplementary Figure 9 Enhancement of amyloid aggregation of ALS-related FUS mutant in HeLa cells infected by SARS-CoV-2 (a) or transfected with N protein (b).

Confocal images of CFP-FUS P525L aggregation puncta in HeLa cells are shown. FUS P525L is visualized by CFP fluorescence. N protein is fused with a Flag tag and immunostained with anti-Flag. pFTAA stains amyloid aggregation. The arrows indicate FUS P525L aggregates. Scale bar, 5 μm. Quantification of the aggregation area per transfected cell in the images is shown (right). Values are means ±S.D., n > 150 cells from 3 repeats. Student's *t*-test.

REFERENCES:

1. Qamar, S., Wang, G., Randle, S.J., Ruggeri, F.S., Varela, J.A., Lin, J.Q., Phillips, E.C., Miyashita, A., Williams, D., Strohl, F., et al. (2018). FUS Phase Separation Is Modulated by a Molecular Chaperone and Methylation of Arginine Cation-pi Interactions. Cell *173*, 720-734 e715.
2. Klingstedt, T., Shirani, H., Aslund, K.O., Cairns, N.J., Sigurdson, C.J., Goedert, M., and Nilsson, K.P. (2013). The structural basis for optimal performance of oligothiophene-based fluorescent amyloid ligands: conformational flexibility is essential for spectral assignment of a diversity of protein aggregates. Chemistry *19*, 10179-10192.
3. Burke, K.A., Janke, A.M., Rhine, C.L., and Fawzi, N.L. (2015). Residue-by-Residue View of In Vitro FUS Granules that Bind the C-Terminal Domain of RNA Polymerase II. Mol Cell *60*, 231-241.
4. Conicella, A.E., Zerze, G.H., Mittal, J., and Fawzi, N.L. (2016). ALS Mutations Disrupt Phase Separation Mediated by alpha-Helical Structure in the TDP-43 Low-Complexity C-Terminal Domain. Structure *24*, 1537-1549.
5. Delaglio, F., Grzesiek, S., Vuister, G.W., Zhu, G., Pfeifer, J., and Bax, A. (1995). NMRPipe: a multidimensional spectral processing system based on UNIX pipes. J Biomol NMR *6*, 277-293.
6. Lee, W., Tonelli, M., and Markley, J.L. (2015). NMRFAM-SPARKY: enhanced software for biomolecular NMR spectroscopy. Bioinformatics *31*, 1325-1327.
7. Dosztanyi, Z., Csizmok, V., Tompa, P., and Simon, I. (2005). IUPred: web server for the prediction of intrinsically unstructured regions of proteins based on estimated energy content. Bioinformatics *21*, 3433-3434.
